# Medical telerobotic systems: current status and future trends

**DOI:** 10.1186/s12938-016-0217-7

**Published:** 2016-08-12

**Authors:** Sotiris Avgousti, Eftychios G. Christoforou, Andreas S. Panayides, Sotos Voskarides, Cyril Novales, Laurence Nouaille, Constantinos S. Pattichis, Pierre Vieyres

**Affiliations:** 1 Nursing Department, School of Health and Science, Cyprus University of Technology, 30 Archbishop Kyprianou Street, 3036 Limassol, Cyprus; 2 Department of Electrical and Computer Engineering, University of Cyprus, 75 Kalipoleos Street, P.O.BOX 20537, 1678 Nicosia, Cyprus; 3 Department of Electrical and Electronic Engineering, Imperial College, South Kensington Campus, London, SW7 2AZ UK; 4 Department of Electrical Engineering, Computer Engineering and Informatics, Cyprus University of Technology, 30 Archbishop Kyprianou Street, 3036 Lemesos, Cyprus; 5 Laboratoire PRISME-Universite d’Orleans, 63 Avenue de Lattre de Tassigny, 18020 Bourges, France; 6 Department of Computer Science, University of Cyprus, 75 Kalipoleos Street, P.O.BOX 20537, 1678 Nicosia, Cyprus

**Keywords:** Telerobotics, Telemedicine, Medical robotics, Surgical robotics, Teleoperation, Telepresence, Telemanipulation, mHealth

## Abstract

Teleoperated medical robotic systems allow procedures such as surgeries, treatments, and diagnoses to be conducted across short or long distances while utilizing wired and/or wireless communication networks. This study presents a systematic review of the relevant literature between the years 2004 and 2015, focusing on medical teleoperated robotic systems which have witnessed tremendous growth over the examined period. A thorough insight of telerobotics systems discussing design concepts, enabling technologies (namely robotic manipulation, telecommunications, and vision systems), and potential applications in clinical practice is provided, while existing limitations and future trends are also highlighted. A representative paradigm of the short-distance case is the da Vinci Surgical System which is described in order to highlight relevant issues. The long-distance telerobotics concept is exemplified through a case study on diagnostic ultrasound scanning. Moreover, the present review provides a classification into short- and long-distance telerobotic systems, depending on the distance from which they are operated. Telerobotic systems are further categorized with respect to their application field. For the reviewed systems are also examined their engineering characteristics and the employed robotics technology. The current status of the field, its significance, the potential, as well as the challenges that lie ahead are thoroughly discussed.

## Background

Telerobotics is considered to be an integral part of the wider field of telemedicine. The ultimate goal of telemedicine is to provide specialized healthcare services over long distances, effectively eliminating the need of physical presence of both the physician and patient in the same location. The possibility of consultation diagnosis, treatment, and medical intervention from a distance, may greatly impact the quality of life of patients located in isolated areas where access to specialized medical services is limited. Telemedicine can virtually bring specialists to areas where medical facilities and experts are not available. Practically, a specialist can examine or operate on a patient at a different geographic location without either of them having to travel. Costs and inconvenience are avoided while improved access to information becomes possible. Moreover, the physician can provide services while at a more comfortable working environment. This method also eliminates the possibility of transmitting infectious diseases between patients and healthcare professionals. Apart from medically-isolated areas, telemedicine is also expected to play a key role in removing barriers to healthcare provision in developing countries, in areas of natural disasters, and war zones where consistent healthcare is unavailable or there is no time to transport a patient to a hospital.

Robotic systems were first introduced in medicine in the mid-80’s and today they make an impact in various medical disciplines including general surgery, neurosurgery, and orthopedic surgery [1–5]. Despite the current challenges telerobotic systems are expected to play a significant role in clinical practice [6]. The first successful telesurgery, named “Operation Lindbergh” was performed using a Zeus robotic system in 2001 [7], where a laparoscopic gall bladder intervention was performed on a patient located in Strasbourg, France while the operating surgeon was located in New York, USA. Despite the fact that the first documented long-distance telesurgery was not conducted until 2001, telesurgical systems were presented much earlier. More specifically, Computer Motion which later merged with Intuitive Surgical Inc., (USA) introduced the automated endoscopic system for optimal positioning (AESOP) system in 1994 [8], a voice-activated arm used in minimally invasive surgery to position and hold an endoscope.

In telerobotic systems, the remote manipulator is controlled from the operator’s site by sending position commands while receiving visual and other sensory feedback information. The local and remote systems are typically referred to as “master” and “slave” systems, respectively, and the overall system is referred to as a “master–slave system”. The remote manipulator is programmed to track the controls of the operator. Figure 2 presents a typical structure of a telerobotic system with additional information specific to the MELODY system for robotically-assisted tele-echography applications (presented in "Long-distance paradigm: the MELODY system" section). Many medical robotic systems employ teleoperation as the major mode of operation; but often the master, also called the expert site, and the slave remote manipulator, also called the patient site, are in fact located in the same room [9, 10]. These systems will be referred to as short-distance telerobotic systems; even in this case, telerobotic systems are effectively split into two sites. First is the local site, which includes the human operator and all components needed to remotely operate the system (monitors, keyboards, joysticks, and other input/output devices). Then is the distant site, which includes the robotic manipulation system and the patient surrounded by the appropriate support personnel. This approach, when applied to surgical interventions, is referred to as telesurgery.

The underlying framework for telerobotics is telepresence. Telepresence requires that the information concerning the remote environment is presented to the operator in a natural fashion, which in turn generates a feeling of presence at the remote site [11]. The actual connection between the master and the slave system is established by telecommunication networks. However, when the distance between the two sites is large, time delays in data transmission might affect the operation of the robotized system that will eventually be reflected on the medical expert’s performance. Telecommunication quality of service and bandwidth capacity are one key point for robotized telemedicine. It can be overcome by a local area network (LAN) in a short distance telerobotic system like the da Vinci, or using a dedicated optic fiber through all the Atlantic Ocean for a successful Lindbergh experiment between USA and France; but this last option cannot be realistic.

The primary objective of this study is to present a systematic review of telerobotic systems and highlight their challenges but also their potential. The rest of the paper is organized as follows. “Enabling technologies for telerobotic systems” section introduces medical telerobotic systems so as to establish the framework of the subsequent review. This section focuses on the telerobotic technology while highlighting associated manipulation, network and video challenges. Then, the case of a short-distance telerobotic system is exemplified by the da Vinci Surgical System. This is followed by a long-distance telerobotic system paradigm relevant to remote diagnostic ultrasound (US) examinations. “Short-distance telerobotic systems” and “Long distance telerobotic systems” sections constitute the main body of the review where telerobotic systems are categorized with respect to their operating distance into two groups, namely short-distance and long-distance systems. “Discussion—future challenges” section discusses the future challenges of telerobotic systems with respect to the key enabling technologies and areas for future developments in medical telerobotics are identified. Some data from the review are presented in the form of tables and charts, the interpretation of which provides a useful overview of the telerobotic field. The last section provides some concluding remarks.

## Enabling technologies for telerobotic systems

### Telemanipulation issues

In the term telerobotic, the prefix “tele”, which originates from the Greek language, implies operation from a distance. However, in the field of robotics, the term telerobotic is commonly used in a wider sense, to imply the existence of a barrier between the operator and the remote environment, which restricts access and limits perception [12]. The barrier can be the actual distance and/or a physical obstruction. In fact, one of the original telerobotic applications involved the handling of radioactive materials. The human operator was situated behind a protective leaded glass window using direct visual feedback to control the manipulator. An analogous paradigm from the field of medical systems, is a robotic manipulator required to deal with physical obstructions, as in the case of robotically-assisted minimally-invasive surgery (MIS) and the natural orifice transluminal endoscopic surgery (NOTES) [13]. In either case, the surgeon typically operates inside a body cavity using laparoscopic vision, while robotic assistance facilitates physical access to that environment. When considering a medical telerobotic system, it is important to identify which type of barriers the system is required to deal with. Based on the abovementioned definition, many of the proposed medical robots (but not all) can be characterized as telerobotic systems. Note that several other terms are often used interchangeably to “telerobotic”: telemanipulation, teleoperation, and remote handling.

Significant interest in medical telerobotics has been documented both for diagnostic (e.g., US diagnostic scan, biopsy) as well as interventional (e.g., therapeutic treatments such as protontherapy, surgery) applications. Most of the proposed systems are application/anatomy–specific (cardiac, orthopedic, neurosurgical, etc.) medical telerobots but there also exist general-purpose ones. The manipulation system effectively extends telepresence beyond the perception of the remote environment, which becomes possible through the available sensory information. As an integral part of the system, the manipulator allows the operator to effectively act in the remote environment, physically manipulate objects, and interact with them thanks to haptic feedback [14]. A medical telerobotic system is capable of performing the required tasks remotely while capitalizing on the inherent advantages of medical robots (steady-hand, accuracy, motion scaling, biomotion compensation, etc.).

Telerobotics applications mostly involve articulated (serial and parallel) robot configurations (mainly customized robots dedicated to the medical application), but other forms were also considered including snake-like robots. Typically, a serial robot consists of a number of links interconnected with actuated revolute, prismatic or other type of joints. At the inboard end of the kinematic chain is the base of the robot and at the outer end is the end-effector (end-tool). For example, the end-effector can be an interchangeable surgical tool. The area that the end-effector can access is referred to as the workspace of the manipulator. A parallel manipulator is typically a mechanical system that consists of several serial chains to support a single platform (the end-effector). In general, serial robots may have a large workspace and good dexterity. The kinematics and control of parallel robots are in general more complex but they provide high-speed displacement and accurate positioning. Among the types of robots considered in telerobotic applications is also included the constant curvature or snake-like robots [15–17], These continuously curving systems are particularly useful when required to access and operate in confined spaces. Actuation often involves continuously bending actuators or the use of tendons. Concentric-tube robots are also included in the same family. They consist of a concentric tubes assembly, with member tubes allowed to telescopically extend/rotate relevant to each other along/about their common axis [16]. Selected members are precurved so that upon extension they assume a curved shape while adjusting the resulting position of the end-effector.

A key characteristic of any manipulation system is the number of available degrees-of-freedom (DOF), which is a design parameter directly associated with the application requirements. A robotic manipulator with many DOF is more dexterous but at the same time the size/weight of the robot increases. Selection of actuation methods is generally not directly related to teleoperation but it rather depends on the application requirements (force, speed, accuracy, etc.) and the operating conditions. Robot manipulators often use electric motors, piezoelectric, hydraulic, and pneumatic actuators. Actuation is an important characteristic of any individual robotic system and it is thus addressed as part of this review.

Herein, telerobotic systems are categorized as “short-distance” and “long-distance” depending on the physical distance separating the operator and the remote manipulator. In the case of short-distance systems, even though the operator’s site is alongside the patient, it is in fact separated from the robot unit, while guidance is based on the acquired images and the transmitted sensory information. In principle, this arrangement enables operating of the manipulator from a larger distance as well. Short-distance systems are mostly associated with the physical barrier case, as already discussed. In the long-distance category, the operator and the manipulator site are geographically separated. The link between them is established either via an existing communication infrastructure or via a dedicated temporary network, which can be either wired or wireless.

The control of telerobotic systems is primarily based on image and video guidance. The involved image acquisition process impacts the portability and transportability of the telerobotic system, while the associated bandwidth demands of the encoded image and video also define to a large extend the telecommunication requirements. Moreover, the image acquisition method may impose further design requirements to the system, as for example in the case of robots operating in the MRI environment, which have to be MR-safe and MR-compatible [18]. Depending on the used imaging method, a telerobotic system can be specific to laparoscopy, ultrasound, computed tomography (CT), magnetic resonance imaging (MRI), and X-ray fluoroscopy.

MRI is characterized by excellent imaging capabilities but accessibility to the patient inside the scanner for real-time guidance of interventions is fairly limited. The use of MR-compatible robots has been proposed to overcome this problem but the development of such robots is challenging because of the high magnetic fields relevant to the operation of the scanner as well as the geometric limitations imposed by the scanner [18]. The telerobotic system provides access to the patient inside the scanner. One example is a teleoperated master–slave interventional system for breast biopsy which was developed by Yang et al. [19]. Under continuous MR imaging the physician uses the master system to operate the slave one, which is located inside the scanner together with the patient. The system has six degrees-of-freedom and MR-compatible actuation combines one piezoelectric motor and five pneumatic cylinders.

Operation of telerobotic systems is commonly based on a man-in-the-loop control approach and involves a master/slave architecture. For articulated robots, the master system often replicates the kinematics structure of the slave system. Such a direct kinematics correspondence between the master and slave devices results in an intuitive operation that simplifies motion handling within the control system. A significant breakthrough in telemanipulation is facilitated by force-reflecting haptic feedback which allows the operator to sense the forces applied by the remote manipulator on its environment. The use of tactile feedback information has also been considered but to a lesser extent. In general, a haptic system’s user interface is composed of bidirectional elements. Auxiliary control functions found in medical robotics, such as motion scaling, biomotion compensation, and hand-tremor filtering, are of particular importance to telerobotics. For example, biomotion compensation will allow a robot to constantly follow the heart’s motion during an intervention. With this capability the physician may operate on a seemingly stationary heart while in reality it is naturally beating. This approach presents a highly desirable alternative to standard arrested-heart techniques, as it was examined in [20] where a predictive feedback control scheme is proposed. In that case, the heart’s motion is measured from ultrasound images and the delay due to image acquisition and processing, which impacts the feedback control loop, is compensated for using a Smith predictor technique.

Control of the basic operation of the manipulator and implementation of the aforementioned functions requires sensory feedback information. Sensors can be either internal or external to the robotic manipulator. The former are directly mounted on the manipulator (e.g., joint position sensors, force sensors) and the latter are separated from the manipulator (e.g., external camera systems) but are also integrated to the control system.

Communication delays and information loss are inherent to long-distance teleoperation. These may severely impact the stability and performance of the controlled system and they pose challenging problems that attracted the attention of the robotics and controls community. A survey that addresses the subject of bilateral teleoperation focusing on several control theoretic approaches was provided by Hokayem and Spong [21]. It covers various methodologies, including passivity-based control, that were proposed to address the aforementioned challenges. Note that passivity-based control is known for its favorable robustness characteristics. Niemeyer and Slotine [22] applied the wave variable concept, an extension to the theory of passivity, to time-delayed teleoperation assuming an unknown but constant time delay. Recently, a special type of force feedback algorithm called projection-based force reflection was examined and experimentally evaluated for the case of a dual-arm haptic-enabled teleoperator system for minimally-invasive surgical applications with communication delays [23].

Advanced control techniques including robust and adaptive control are particularly relevant to bilateral teleoperation systems. Robust control is capable of preserving stability and performance despite uncertainties or disturbances affecting the system. In general, adaptive control has the ability to adapt to controlled systems with unknown or varying parameters. Certain model-based adaptive controllers have the ability to extract parameter information about the controlled system and then use it towards improving control performance. One such example is the case of [24], where an adaptive control scheme is proposed to deal with both dynamic and kinematic uncertainties regarding a remote manipulation system while communication delays are also taken into account.

Robotic systems are often categorized into “autonomous”, “semiautonomous”, and “teleoperators”. Fully autonomous systems are essentially beyond the scope of telerobotics which primarily involve human-in-the-loop control. Using a fully autonomous medical robot, the physician expertise (and responsibility) disappears and the added value is not so obvious nowadays. A complementary robotic action to assist or to train the expert is preferable. An example of semiautonomous operation is the case of stereotactic interventions, in which planning can be carried out based on preoperative images at the operator’s site, while the actual plan is executed by the remote telerobot. Naturally, the involved registration procedure has to be handled locally at the patient’s site. Another paradigm of semiautonomous operation is the case of a remotely-controlled robot with some auxiliary control functions (e.g., biomotion compensation) handled autonomously by the robot itself. A trend towards an increasingly active role assigned to the robotic system does exist in order to help improve the efficiency of operations.

Telerobotic systems may involve fixed installations in hospitals, systems installed on mobile platforms (e.g., ambulances, trains, ships, airplanes) as well as fully portable (and even handheld) systems. Systems belonging to the last two categories effectively extent the scope of telerobotics to the wider field of mobile-health (m-health) systems and services [25]. As a result, features such as mobility and transportability become important to many telerobotic systems. These features are determined by factors including the size and the weight of the equipment, the mounting options, the power demands and the telecommunication requirements, as well as the support staff needs.

### Network issues

In order to develop a network-based telerobotic system which is bilaterally controlled in real time, thus giving to the user a sense of being present in the remote environment, it is necessary to find ways to overcome all limitations posed by the network substructure-system. In a telerobotic system, the master station controls a remote robot by sending position commands and accepting force and visual feedback, in addition to information on slave robot position and status [26]. Typically, in any telerobotic system the communication system must support the three types of data flows described below.

1. *Real-time control data*, means that the (or a part of the) control loop of the robot pass through the communication link. So network quality and delay are preponderant. Typically in medical robotics, robot servoings are localized on the patient site, at the end of the connection. But when force feedback is necessary at the expert site (haptic control), the control loop must pass through the net. The bi-directional data flow is at a constant rate and comprised of small packets. The issuing rate corresponds to the sampling frequency of the control loop, and the quality of the force feedback perceived at the master station depends on it. Moreover, as the afore-described closed-loop process is affected by the loop delay, the time needed to transfer each packet restricts the system’s performance with respect to closed-loop control bandwidth. In addition, possible data packet losses, more likely to occur in the air interface due to phenomena like multipath propagation, Doppler effect, and noise, could influence the loop in an undesirable way.
2. *Medical video stream* This is the video stream that is transmitted from the slave to the master. The responsible video encoder determines the characteristics of this flow in terms of bit rate demands and data packets’ size. Various video streaming solutions are available over a digital communication media, such as traditional RTP protocol based streaming and emerging adaptive HTTP streaming, while lower-quality videoconferencing is typically used for ambient video transmission. Video quality may be traded with bit rate requirements, and as most of the available standards have been designed for non-reliable communication media, they are tolerant against limited data losses. In any case, diagnostically lossless video communication (i.e., the clinical capacity of the medical video is not compromised during transmission) must be preserved.
3. *High-level management data* This covers data from the slave to the master site, such as setting the control loop’s sampling frequency, resetting the system, etc. This flow is negligible in terms of network resources usage, as it mostly consists of small packets, sent sporadically during the robot operation. Nevertheless, this data flow requires a reliable data connection such as TCP/IP.

In nearly all teleoperation systems there is some time delay while operating, posing a significant issue on the synchronization and operation of the exchanging commands, and hence degrading system stability and performance [27]. When the master and slave are close to each other, this delay is usually negligible and can be compensated for with the appropriate controller. On the other hand, if the master and slave are located at a long distance from each other, the time delay is no longer negligible, especially for bilateral teleoperation systems. In multimodal telerobotic systems, the delay problem does not exclusively arise from the latency and jitter in the communication, but is also due to the temporal inconsistency between the various sensory modalities.

Varying time delays in the communication links can be distinguished in the mean end-to-end delay (latency) and its variation (jitter). Both latency and jitter are attributed to the communication’s system infrastructure, and primarily to the air interface. In general, it is very difficult to define a comprehensive, analytical communication model. The latter is due to the fact that packets routes are allocated dynamically depending on the network load and given that the air interface is shared by multiple users with diverse demands, it is very difficult to map. Moreover, network nodes may have different routing policies, throughput, buffering and queue management, and hence data packets are subject to different treatment policies at each node they traverse.

In cases where the number of data packets exceeds the available bandwidth, congestion occurs. The latter factors affect the delay in the data packets exchanged between two computers. That said, recent advances in wired and wireless infrastructure, have significantly reduced the incorporated latencies, providing for more reliable telerobotic operation. More specifically, 100 ms latency found in EDGE systems (Rel’4) has dropped as much as ten times to 10 ms in LTE-Advanced wireless systems [28] (see Table 1). With robustness and reliability still debatable, theoretical delays facilitated by the underlying infrastructure, are well below the 250 ms delay that is perceivable in terms of visual feedback as documented in [29]. Based on operator’s/expert motor commands and proprioception, a delay of 250 ms in the visual feedback is easily recognized by the human operator.

**Table 1:** Wireless network technologies and user perceived data transfer rates

Technology	Network theoretical data transfer rates	User typical data transfer rates
2G-GSM (early 1990s)	9.6 to 115 kbps	About 10 kbps
2.5G-GPRS (2001)	9.6 to 171.2 kbps	Between 30 and 50 kbps
2.5G-EDGE (2003)	9.6 to 384 kbps	Between 75 and 135 kbps
3G-UMTS (Release 99, 2001)	144 kbps to 2 Mbps	Between 200 and 300 kbps
3.5G-HSPA (Rel. 7, 2007)	DL: 14.4 Mbps	DL: 1 to 4 Mbps
(HSDPA, Rel. 5, 2005)	UL: 5.76 Mbps	UL: 500 kbps to 2 Mbps
(HSUPA, Rel. 6, 2008)		
HSPA + (Rel. 7, 2007)	DL: 21.6 Mbps	DL: ~2 to ~9 Mbps
	UL: 11.5 Mbps	UL: 1 to 4 Mbps
HSPA + (DL: 64 QAM, UL: 16 QAM, dual carrier, 10 + 5 MHz)	DL: 42 Mbps	DL: 3.8 to 17.6 Mbps
	UL: 11.5 Mbps	UL: 1 to 4 Mbps
3.5G-Mobile WiMAX (IEEE 802.16e, 2005)	DL: 46 Mbps	DL: UL: 1 to 5 Mbps
	UL: 4 Mbps	
3.9G LTE (Rel. 8, 2008)	DL: 300 Mbps (20 MHz)	DL: 6.5 to 26 Mbps (10 MHz)
	UL: 71 (20 MHz)	UL: 6.0 to 13.0 (10 MHz)
4G-LTE-advanced (Rel. 10, 2010)	DL: 1.2 Gbps	TBD
	UL: 568 Mbps	
4G-WirelessMAN-advanced (IEEE 802.16 m, 2010)	DL: > 1 Gbps	TBD
	UL: > 100 Mbps	
5G by 2020?	TBD	TBD

Table based on [28]

*UL* uplink, *DL* downlink *TBD* to be defined

There were many studies reported in the literature focusing on time delay in teleoperation control systems [30]. Hokayem and Spong in [21] gave a historical survey on teleoperated control systems strategies. Results presented in [22, 31] considered that the communication time delay between master–slave teleoperators is constant. Most of the solutions proposed in the literature are based on the assumption that human and environment input forces to the master and slave robots are passive which may be difficult to satisfy in a real-time application [32–34].

Bearing in mind the aforementioned, a network-based bilaterally controlled telerobotic system must be designed addressing the following important issues: (a) ensuring the stability of the control loops over the communication network’s varying state, and (b) implementing a strict resource allocation policy, as the typical case is to use the same physical connection for all data flows. Unpredictable behavior exhibited by both wired and wireless packet-based networks in terms of communication delay, jitter, and packet data losses—throughput, pose a significant challenge. Obviously, by overcoming or minimizing the effects of the abovementioned network limitations, it would be feasible to design more reliable and efficient real-time telerobotic systems.

### Medical video communication issues

Medical video communication, and more specifically wireless medical video communication, poses significant challenges in medical telerobotic systems. Medical videos dominate over robot control data and other biosignals, both in terms of bandwidth requirements as well as processing needs. Given that this is a crucial component—central to the success of telerobotic systems—used for guiding teleoperated processes and providing remote diagnosis, an optimum trade-off that both satisfies the medical video quality requirements while not compromising the teleoperation process via over flooding available bandwidth, is the primary objective of such systems and services.

This goal was widely investigated in the literature over the past decade [35]. Documented approaches are tightly coupled with associated advances in enabling technologies, namely video compression and wireless infrastructure. Video compression standards are responsible for compressing the acquired video so that it is suitable for transmission over the current best available wireless network (i.e., resulting bitrate demands conform to the wireless infrastructure’s available upload data rates). At the same time, clinical quality cannot be compromised by the compression process, as this would result in insufficient data in the communicated video for the remote medical expert to provide a confident diagnosis. Failing to do so will significantly affect the reliability and quality of service of the system and hence its clinical usage. Moreover, video compression standards play a key role—in conjunction with the employed device’s processing capabilities—in materializing real-time encoding and decoding. The latter is of primary importance in medical telerobotic systems as no other mode of operation is feasible other than real-time operation. In terms of wireless infrastructure, available upload data rates directly impact the quality of the communicated medical video. The more the bandwidth, the higher the clinical image (less compression) and the room to jointly accommodate robot control data (see Table 2). Toward this end, the control operation is highly affected by the underlying wireless infrastructure, as the responsiveness of the robot controls and manipulations are essentially dictated by the wireless network’s involved latencies. The lower the latency, the lower the delay between the remote ends and hence, the more responsive the telerobotic system.

**Table 2:** Ultrasound video bitrate savings of different video coding standards in time

Encoding	Bit rate savings relative to			
	H.264/MPEG-4 AVC HP (%)	H.263 CHC (%)	MPEG-4 ASP (%)	MPEG-2/H.262 MP (1995) (%)
HEVC MP (2013)	33.2	54.6	58.3	71
H.264/MPEG-4 AVC HP (2003)		33.2	37.7	56.8
H.263 CHC (2000)			7.5	32.4
MPEG-4 ASP (2000)				27.4

Table originally published in [143]

Bearing in mind the aforementioned, wireless medical video communication systems evolved to diagnostically-driven systems [35]. The term diagnostically-driven was coined to highlight the algorithmic approaches designed to improve the diagnostic capacity of the communicated medical video. The latter is materialized via adopting both the encoding and transmission process to the underlying medical video modality. Exploiting the video’s properties allows the development of efficient, context-aware algorithms that in turn lead to increased clinical image quality. One of the most prevailing approaches is diagnostic region of interest (d-ROI) based systems. The key concept is that certain regions in a medical video contain more clinical information than other video regions. Based on this observation, quality levels during compression can be allocated with respect to the video region’s diagnostic significance [36]. In this manner significant bandwidth gains are achieved by applying higher compression levels on the background, non-diagnostically important regions. In a similar way, these d-ROI can be protected more strongly during wireless transmission using stronger forward error correction modes, error resilience techniques, and retransmission mechanisms. Despite the fact that d-ROI systems tend to be medical video modality specific, this approach was adopted for a plethora of wireless medical video communication systems [35].

The emergence of the new high efficiency video coding standard (HEVC) and 4G and beyond (towards 5G) wireless networks are expected to play a catalytic role in medical video communication systems and to be capitalized within the context of telerobotic systems. HEVC provides higher compression efficiency and parallel processing technologies. Linked with significant upgrades in available data transfer rates in the uplink and lower latencies in 4G systems and beyond, responsive telerobotic systems in clinical practice, facilitating wireless medical video communication that will rival the quality of in-hospital examinations, are envisioned. Low-delay high-resolution medical video transmission of high diagnostic capacity ultrasound video has been already highlighted in the literature for in-ambulance telemedicine applications [37]. The challenge is then adapting to varying wireless networks conditions, especially during in-ambulance remote examinations as in the case-study described in “Short-distance paradigm: the da Vinci system” section. Real-time adaptation to varying bandwidth availability while conforming to medical video clinical requirements and securing low-delay robot controls communication is critical to the success of telerobotic systems. As a general rule, in any telerobotic system, appropriate provisions should be installed that prioritize the communicated robot controls, with respect to the transmitted medical video. The latter can be accomplished using multi-objective optimization. Such a framework, which jointly optimizes medical video quality to facilitate remote diagnosis, bandwidth demands to match the available data rates, and encoding time to conform to real-time requirements was proposed in [38]. The proposed approach is scalable and technology independent, and thus can be employed in a plethora of telerobotic systems, where the constraints imposed by the underlying medical video modality, the employed device and video compression standard, and the wireless infrastructure, can be abstracted into system parameters.

### Short-distance paradigm: the da Vinci system

**Fig. 1:**
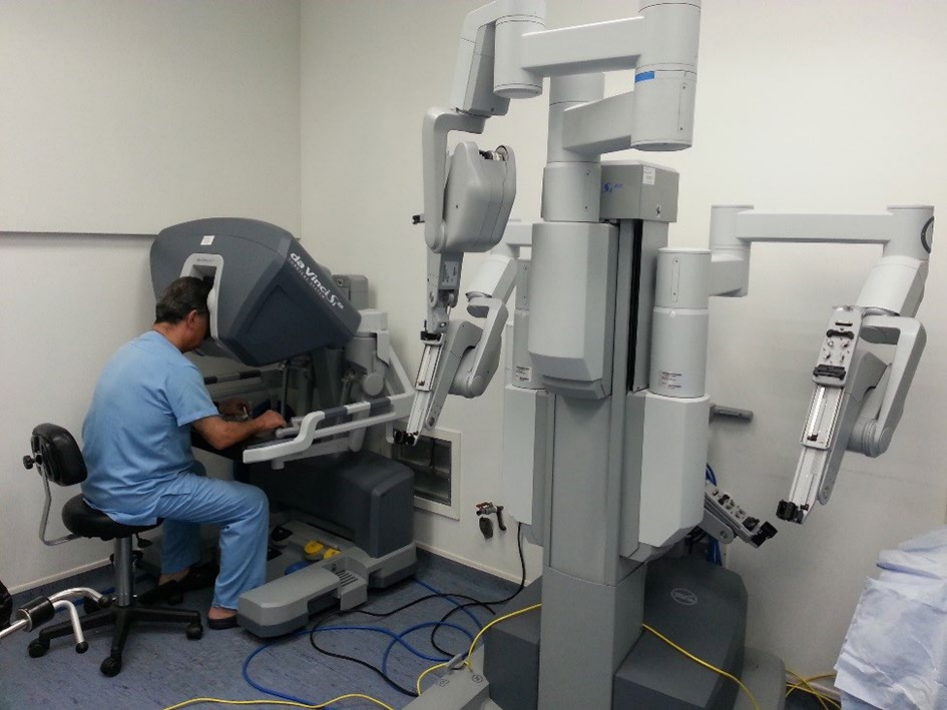
The da Vinci® surgical system [141]

A most advanced telemanipulation system called the da Vinci system (Intuitive Surgical, Inc.) was developed for minimally invasive laparoscopic surgery. It operates on the basis of a master–slave control concept. The da Vinci system (Fig. 1) is considered a landmark development in robotic surgery together with AESOP and ZEUS [8]. Da Vinci comprises of two main units. First is the surgeon’s ergonomic console unit which includes the display system, the surgeon’s user interface and the controller. The second unit comprises of four slave manipulators, three for telemanipulation of surgical instruments (EndoWrist Instruments) and one for holding the endoscopic camera. The system provides the medical expert with a realistic operating environment that includes a high-quality stereo visualization and a man-machine interface that directly transfers the doctor’s hand gestures to the instrument tip movement inside the patient [39]. The doctor is presented with magnified stereoscopic images through a 3D display, restoring hand–eye coordination and rendering instinctive matching with manipulations [40]. The latest generation of the da Vinci system also provides for a dual console option that allows two surgeons to work collaboratively. This facilitates more efficient training of surgeons, especially those unfamiliar with robotic-assisted surgery. The da Vinci system is currently used for a variety of surgical interventions: general, thoracic, cardiac, colorectal, gynecology, urological, etc. The technical aspects of the da Vinci robotic system were reviewed in [40].

The concept of the da Vinci theoretically allows remote teleoperation in long distance, but the previous versions of the robot used a proprietary short-distance communication protocol through optic fiber to connect the master and the slave stations. The latest versions of the system facilitate further displacement of the two units. In 2005, Telemedicine and Advanced Technology Research Center (TATRC) presented collaborative telerobotic surgery (four nephrectomies on a porcine model) with modified da Vinci consoles, being able to overtake the control of one with the other through a public internet connection [41].

The extensive clinical use of da Vinci surgical system is reflected by the big number of relevant published articles. An extensive list of references compiled by the robot manufacturer [42] contains published studies and reports examining the use of the system. A report on robotic surgery focusing on the use of the da Vinci Surgical System [43] is indicative of the wide range of application procedures and documents the surgeons’ experiences. Specifically, it covers urological procedures (radical prostatectomy, radical and partial nephrectomy, radical cystectomy, pyeloplasty), cardiac procedures (mitral valve repair), and gynaecological procedures (total hysterectomy for endometrial cancer staging, radical hysterectomy for cervical cancer, hysterectomy for mixed benign conditions, myomectomy, tubal re-anastomosis, sacrocolpopexy). In general, when compared to conventional surgical approaches robotic surgery was considered advantageous in terms of reduced blood loss and postoperative pain, shorter hospitalization, and quicker return to daily activities. The risk of severe complications is not increased. The operative times were found equal to or longer compared to conventional approaches but robot-assisted surgery was considered more comfortable for the operating surgeon because of the ergonomics of the system. Specific concerns were expressed regarding the initial cost of robot acquisition but also the ongoing costs for maintenance and training. Among the drawbacks expressed was included the increased set-up times before the surgery can begin. It was also stressed the need for an appropriately trained team consisting of the surgeons, nurses, anesthetists and technicians.

### Long-distance paradigm: the MELODY system

**Fig. 2:**
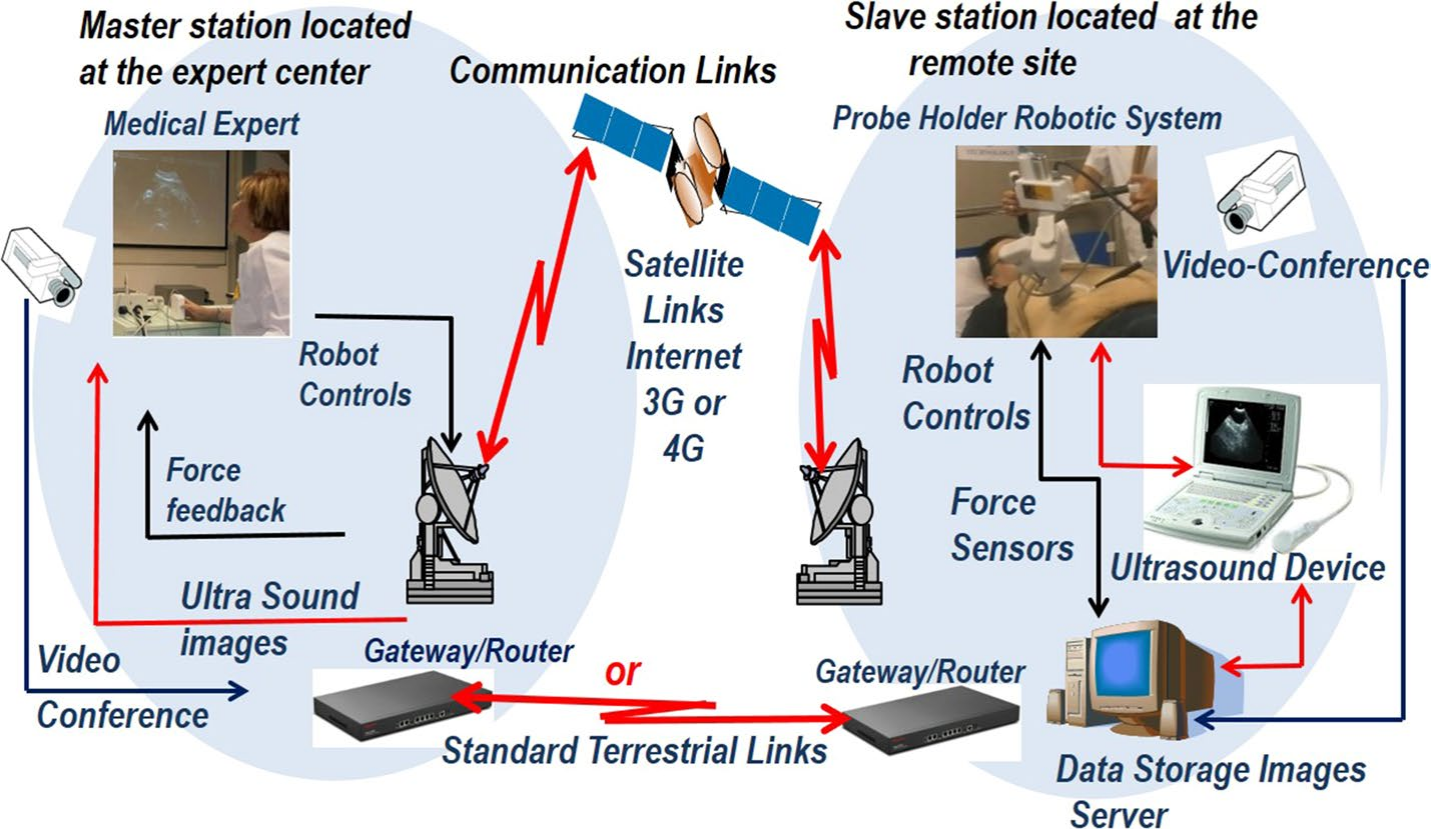
Robotized tele-ultrasound using the MELODY system [54]

Ultrasound is an imaging modality that plays a significant role in medical emergency and surgical decision-diagnosis. To compensate for the limited availability of ultrasound experts in remote and/or isolated areas, the use of robotic telemedicine systems is gaining attention [44–48]. Toward this direction, a tele-ultrasound portable robot prototype was developed in the Laboratory of Vision and Robotics (LVR, renamed PRISME Laboratory since 2008) at the University of Orleans in 1998 (France). Based on PRISME preliminary research work and on the Teresa system [49], AdEchotech SME (France) is now commercializing MELODY tele-ultrasound robotized system; it consists of three main parts: the expert system (master station), the patient system (slave station), and the communication link that enables data exchange between the two stations. MELODY’s system architecture appears in Fig. 2.

At the patient’s site, the US probe is held and manipulated by the 3DOF robotic system following a master/slave control approach. The lightweight robot itself is held and positioned on the patient by a paramedic. A strain gauge force sensor, embedded in the probe holder, measures the contact force between the real probe and the patient’s skin. The robot controller enables to limit this force to 20 N for the patient’s comfort and safety. If a classical force feedback control is assumed [50], the update rate of the robot control data is up to 1 kHz without loss or jitters, to render the teleoperation scheme “transparent” [51].

In this example, the robot is open-loop-controlled over the communication link but locally closed-loop-controlled on the patient site. This approach removes the control instability problem. Furthermore, it was shown that the design of this robot enables the use of a geometric control [52]. In other words, the robot can be accurately positioned by using a closed-form, fast-computable geometric model. This provides a major safety advantage: when there is a communication cut, the robot only remains still at its last set-point position. This characteristic allows the use of “lighter” network protocols without a data-recovering feature in the case of loss like for instance transmission control protocol (TCP), which has a heavy data frame, overhead and may cause long unpredictable delays for lost data recovery. Faster but more hazardous protocols can be used such as user datagram protocol (UDP) or real-time transport protocol (RTP) to enable the ultrasound specialist to perform a live remote echography [53].

At the master site, the medical expert moves a fictive ultrasound probe as required for an echographic examination. This hands-free input device allows the medical expert to perform natural medical gestures as in conventional conditions. These sensors provide the set points for the robot control. On its basic structure, the fictive probe is a passive device that is spring mounted, making the operator feel as though he/she is applying pressure on a patient’s body. An upgrade patented version of this device is fitted with a force sensor and a 6D localization magnetic sensor giving the attitude and position of the fictive probe in real time; this system also integrates an actuator that can be controlled to render to the expert the effort sensed by the robot end effector on the patient.

The expert’s hand motion is sensed and the information is transferred in real time via a communications link (terrestrial or satellite) to the robot’s site, where the manipulation system replicates this motion. The system also maintains the appropriate contact pressure with the patient.

In addition to the US video, the expert receives information about the current robot position/orientation and the applied contact force. The remote probe-holder robot receives the command signals produced by the expert who also adjusts the required contact force. The telerobotic system is enhanced by integrating a videoconferencing system which is used for visual and auditory interactions between the expert, the patient, and the assistant. The connection between the two remote sites for data/image transmission is performed using a TCP connection. When a robot control loop closes through the network, data are then transmitted using the UDP protocol.

As this is a multimodal application, four kinds of data are transmitted and can be identified:

1. *Synchronization flags* These data are small bidirectional byte packets that are transmitted asynchronously. As their name implies, these flags are used to synchronize the sequences of actions of the application on both the expert and the patient sides. For instance, these flags are necessary for the initialization phase.
2. *Robot control data* This control data-flow is bidirectional and is used for Feedforward (expert site to the patient site) for the robot set points, and FeedBack (patient site to the expert site) for the robot status.
3. *Videoconference data* For a friendly usage and to allow the specialist to communicate with his/her patient and assistant staff, a videoconferencing channel is necessary. These data are throughput-demanding but are not critical; consequently, a low quality of images and sound, provided by standard videoconferencing protocols, can be tolerated.
4. Ultrasound video: transmitted from the expert site to the robot site.

**Fig. 3:**
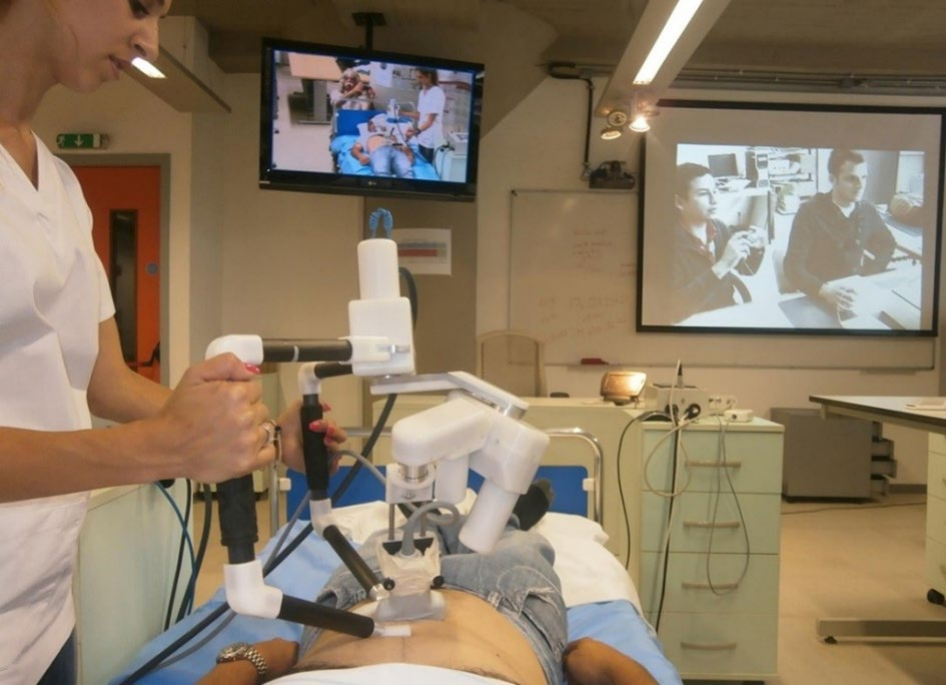
WORTEX 2012 [54] experiment demonstrated the intercontinental feasibility of remote robotized tele-echography in a range of cultural, technical and clinical contexts

An extensive evaluation program termed WORTEX 2012 [54] was carried out in 2012 to demonstrate MELODY system’s capacity to accommodate remote, teleoperated US examination (Fig. 3). It included intercontinental trials involving heterogeneous socio-cultural, technical, clinical, and governmental networks. Five geographical sites and four countries were chosen to serve as remote expert and patient sites and accommodate different, global tele-echography interventions. Experimental evaluation investigated two tele-echography scenarios, namely “from main hospital to a local hospital” and “from a main hospital to an isolated location”. The feasibility of intercontinental tele-echography in a range of clinical contexts was successfully demonstrated. The key benefits of adopting remote, tele-operated ultrasound examination were identified as (i) access to expert healthcare for remote communities globally, (ii) improved patient outcomes, and (iii) cost-effective service delivery. On the other hand, the primary difficulties limiting wider adoption of such technologies were identified such as (i) gaining policy maker and health service management commitment, and (iii) securing the necessary investment [54].

## Short-distance telerobotic systems

This study provides a systematic review of medical telerobotic systems based on publications of the past decade, and more specifically between the years 2004 and 2015. It aspires to document the significant advances achieved in this area, discuss prevailing concepts, and highlight future challenges. For this purpose, the review encompasses not only clinically approved, commercial systems but also experimental approaches. Telerobotic systems are classified into short-distance ones (master and slave systems located in the same room) and those operating in long physical distance. The current section is dedicated to the review of short-distance systems, which are tabulated in Table 3 and it is by no means exhaustive. Long-distance ones are reviewed in “Long distance telerobotic systems” section. The considered short-distance telerobotic systems are further categorized with respect to their application field into: general surgery, eye surgery and ENT surgery, neurosurgery, cardiac and thoracic, gastrointestinal and colorectal surgery, urologic, and spinal intervention systems. The table also includes the development stage of each system.

**Table 3:** Summary of short-distance telerobotic systems

	Name	Mechanical design	Application area	Status	References
1	Teleoperated needle insertion robot	Serial	General surgery/intervention	EXP	[55]
2	MR-guided thermotherapy	Parallel	Thermotherapy	EXP	[56]
3	Surgical system for breast biopsy	Parallel	Breast biopsy	EXP	[57]
4	CT-guided needle-placement robot	Serial	Diagnostic and therapeutic needle placement	EXP	[58]
5	Medical robot for MIS	Serial	Surgery (minimally invasive)	EXP	[59]
6	SOFIE	Serial	Surgery (laparoscopic and thoracoscopic)	EXP	[62, 60]
7	Telelap ALF-X	Serial	General surgery	EXP	[63, 64]
8	Al-Zahrawi surgical system	Serial	General surgery	EXP	[65]
9	Telerobotic system for minimally invasive surgery	Snake-like	General surgery (throat and upper airway)	EXP	[66]
10	IREP robot	Snake-like	Surgery (Single Port Access)	EXP	[67]
11	SPS manipulator	Serial	Surgery (Single Port Endoscopic)	EXP	[68]
12	SPRINT	Serial	Surgery (Single-Port Laparoscopic)	EXP	[69]
13	Endoscopic prototype telerobotic system	Serial	Surgery (transluminal endoscopic surgery)	EXP	[70]
14	Robotic (NOTES) device	Serial	Gastrointestinal (NOTES)	CLIN	[71]
15	RVIR robot (vascular interventional robot)	Supporting manipulator/catheter navigator	MIS	EXP	[72]
16	Telerobotic-assisted bone-drilling system	Linear/rotational stage	Surgery (orthopaedics)	EXP	[73]
17	Trauma pod	Serial	General surgery	EXP	[74]
18	Slave manipulator with roll-pitch-roll wrist	Serial	General surgery	EXP	[75]
19	Robotic system for corneal keratoplasty	Cartesian	Eye surgery	CLIN	[76]
20	Snake-like robot for upper airway surgery	Snake-like	Surgery (throat and upper airways—ENT)	EXP	[66]
21	Robotic system for transnasal surgery	Snake-like	Transnasal surgery—larynx and airways	EXP	[77]
22	LANS	Cartesian	Neurosurgery	EXP	[80, 81]
23	NeuroArm	Serial	Micro-neurosurgery and stereotaxy	CLIN	[82, 83]
24	MRI guided neurosurgery	Serial	Neurosurgery	EXP	[84]
25	Master–slave robotic platform for micro-neurosurgery	Spherical	Neurosurgery	EXP	[85]
26	Heart surgery robot	Spherical	Cardiac and thoracic	EXP	[87, 144]
27	MIRS (MIRoSurge)	Spherical	Cardiac and thoracic	EXP	[88, 90]
28	HIFU for kidney ablation	Cartesian	Cardiac and thoracic	CLIN	[89]
29	MIS laparoscopic robot	Spherical	Cardiac and thoracic	EXP	[91]
30	Robotic forceps manipulator	Serial	Cardiac & thoracic	EXP	[92]
31	ZEUS MI robotic lung brachytherapy	Serial	Cardiac and thoracic	COM	[93, 94]
32	da Vinci	Serial	Cardiac and thoracic	COM	[39–41]
33	Heartlander robot	Cable-Driven	Cardiac and thoracic	CLIN	[95]
34	Sensei and artisan	Snake-like	Cardiac and thoracic	COM	[97, 98]
35	MARVEL		Cardiac and thoracic	EXP	[99]
36	Creeping colonoscopy robot	Worm-like locomotion	Gastrointestinal	EXP	[100]
37	Robotic magnetic steering and locomotion of capsule	Serial	Gastrointestinal	EXP	[102]
38	GI robot with active motion	Legged locomotion	Gastrointestinal	EXP	[101]
39	Wireless GI robot	Worm-like locomotion	Gastrointestinal	CLIN	[103]
40	Prostate brachytherapy robot	Serial	Urologic	EXP	[105]
41	MRI guided prostate robot	Cartesian	Urologic	COM	[106]
42	Pneumatic robot for prostate	Spherical	Urologic	EXP	[107]
43	SpineNAv	Serial	Spinal intervention	EXP	[110, 112]
44	CoRA	Closed-loop	Spinal intervention	EXP	[108]
45	MINOSC	Cable-driven	Spinal intervention	EXP	[111]

*EXP* experimental, *COM* commercial, *CLIN* clinical

### General surgery/intervention

**Fig. 4:**
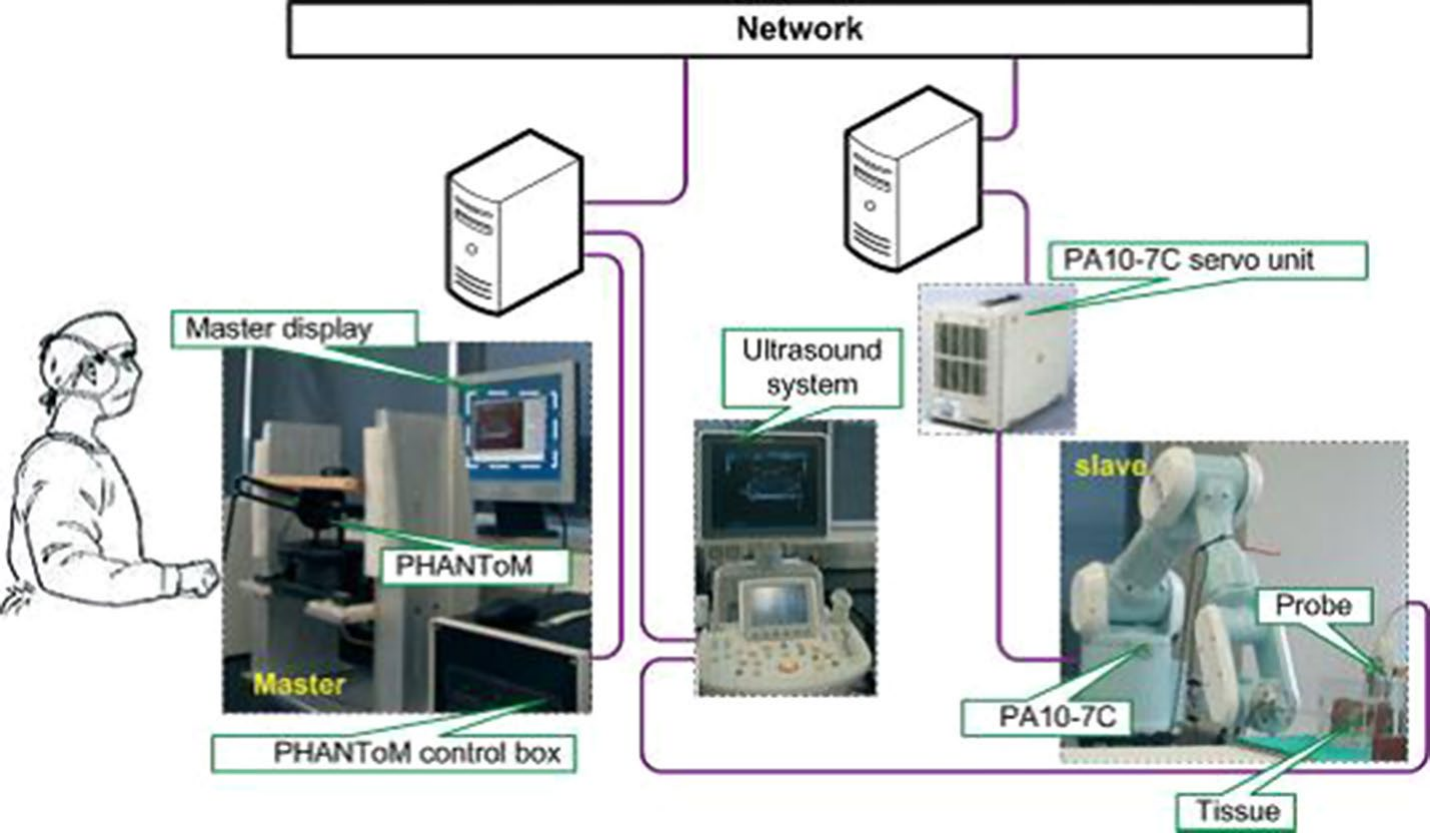
*Block diagram* of the master–slave experimental set-up for needle insertion. Needle targeting can be carried out using images and preoperative planning tools (e.g., stereotactic approaches) or under real-time guidance using a suitable imaging modality (e.g., US). Reprinted with permission from [55]

A class of diagnostic and therapeutic applications involve the placement of needles (biopsies, aspirations, ablations), which can be effectively performed by robots with specially-designed end-tools. Needle targeting can be carried out using images and preoperative planning tools (e.g., stereotactic approaches) or under real-time guidance using a suitable imaging modality (e.g., US). A master–slave robotic system for needle insertions under US guidance was presented by Abolhassani and Patel [55] (Fig. 4). The master station uses the provided position and force feedback measurements from the end-point. The slave station has 7 DOF, three of which are passive while the remaining four are actively controlled. The needle holder is equipped with a force/toque sensor and a needle with a beveled tip. In order to monitor the needle tip position in three dimensions (3D) during an insertion, a sensor coil is inserted inside the needle and is tracked by a magnetic tracking system.

Among the modalities considered for real-time needle placement applications is also MRI. Needle-delivered therapies and diagnostic procedures such as biopsies, aspirations, local drug delivery, implantation of radioactive brachytherapy seeds at a tumour site and ablations (e.g., RF ablation, cryoablation) for the treatment of tumours can be carried out effectively under MRI guidance. An example is the MR-compatible robot presented by Ohara et al. [56] which operates in open MRI scanners so that images can be used for guiding and monitoring a thermal therapy of liver tumor. The robot has 3 DOF and can control the needle orientation with a kinematics structure combining a five-bar linkage and a gimbal mechanism.

A specialized teleoperated master–slave surgical system for performing breast biopsy under continuous MRI guidance was proposed in [57]. The MR-compatible slave robot is actuated with five pneumatic cylinders and one piezoelectric motor allowing operation inside the MRI bore. The slave robot consists of a three-link parallel mechanism, an X–Y motion stage, and a needle driver to achieve the desired arbitrary needle orientation and position configuration under the space of the breast coil. The master device has a similar kinematic structure as the slave robot and it is used to adjust the needle orientation prior to performing a needle insertion. The slave and the master systems have a dedicated control PC with communication based on ethernet technology and TCP as transport protocol.

**Fig. 5:**
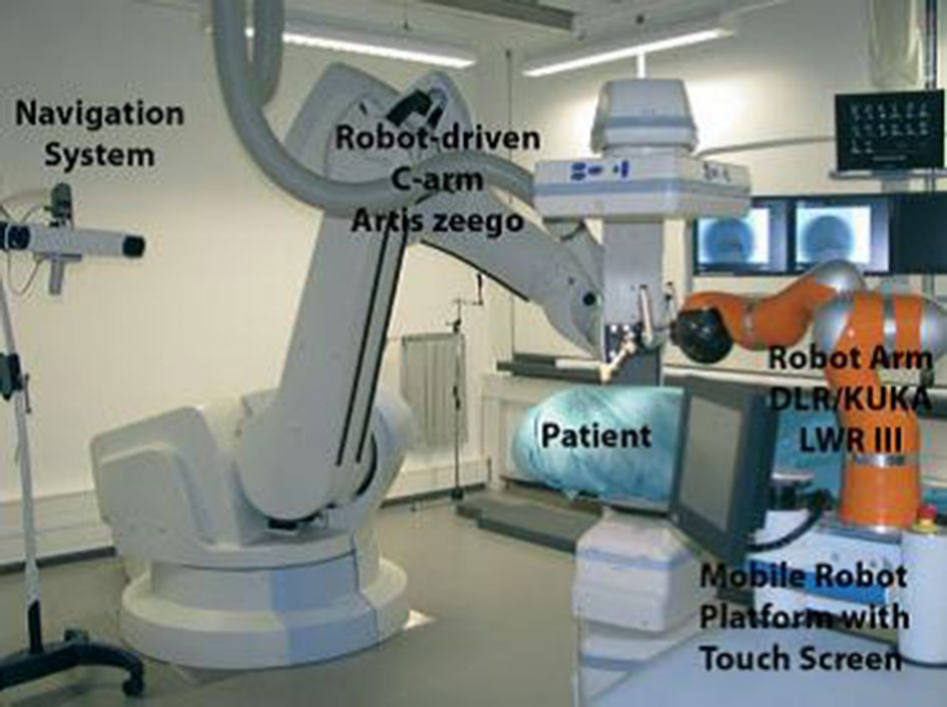
System for CT-guided robotically‐assisted interventions. Reprinted with permission from [58]

The use of CT guidance for robotically assisted percutaneous diagnostic and therapeutic interventions using needles was presented in [58] (Fig. 5). The system consists of a lightweight robot mounted on a mobile platform, a robot-driven angiographic C-arm CT system and a navigation system. It can be positioned and moved around the patient’s table. The robotic system controls the needle alignment by means of intraoperative navigation according to patient-specific planning. For this purpose the image data, 2D projections and 3D volumes are transferred to the robotic planning station. The surgeon can choose the target and an appropriate entry point based on the images. Both the robot and the patient positions are tracked with an optical tracking system which is fixed to the C-arm.

**Fig. 6:**
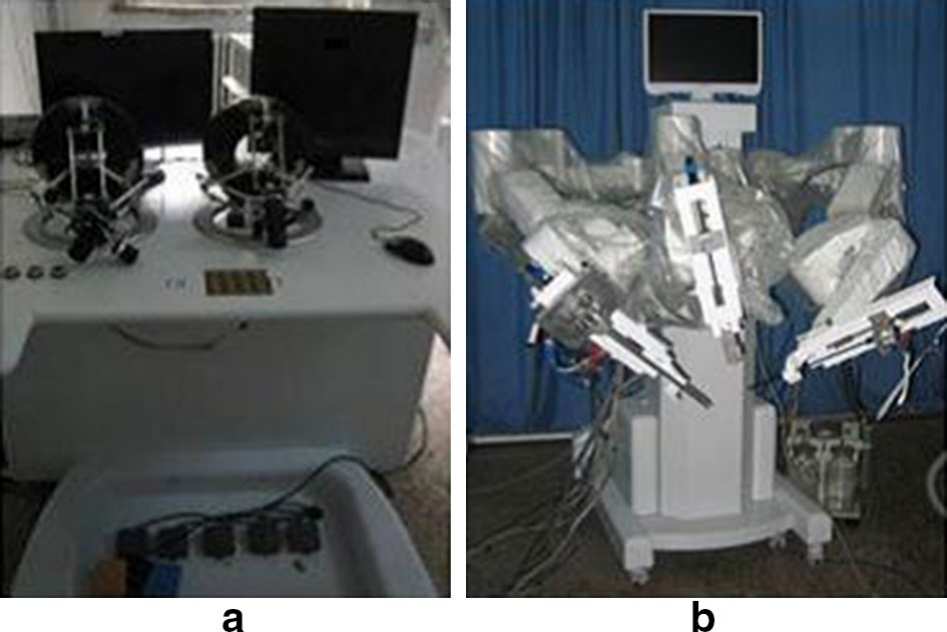
Medical robot for MIS: **a** surgery console; **b** robotic arm cart. Reprinted with permission from [59]

Minimally-invasive surgery (MIS) has been a major development in clinical practice [13]. Various surgical interventions are carried out through small incisions on the body (e.g., abdominal wall) providing percutaneous access for the surgical instruments and laparoscopic cameras. Thin tubes called trocars are placed which serve as gateways for the instruments to pass. This method offers significant advantages including reduced trauma to the body, less risk of infection, faster recovery and minimal scarring. Recognizing the advantages of robots in MIS (namely accuracy, steady-hand, hand-tremor filtering, motion scaling, biomotion compensation, avoidance of reverse-hand motion) various telerobotic systems were developed for this purpose. But MIS adds a specific constraint for the robot motion: the tools inserted inside the body cannot be shifted in the trocar. The motion of the terminal tool must respect this remote center-of-motion, either by its control, or by the native mechanical design of the robot. Often, these systems consist of multiple arms that can work collaboratively, as in the case of [59]. The specific robot, shown in Fig. 6, consists of three main components: the surgeon’s console, the robotic arm cart and the surgical instruments. The doctor’s console provides the computer interface between the doctor and the robotic arm cart. The motion of the robotic arms can be managed by the medical expert via master manipulators, a foot control as well as hand gestures. The robotic arm cart comprises of three individual robotic arms, which can be rolled to and from an operating table. The first robotic arm holds tools like a grasper or a scissor. The second arm holds a laparoscope providing a visual display of the operation field, and the third arm holds a high-frequency electric knife or ultrasonic scalpel, which can be used to cut tissue or a tumor without bleeding [59].

**Fig. 7:**
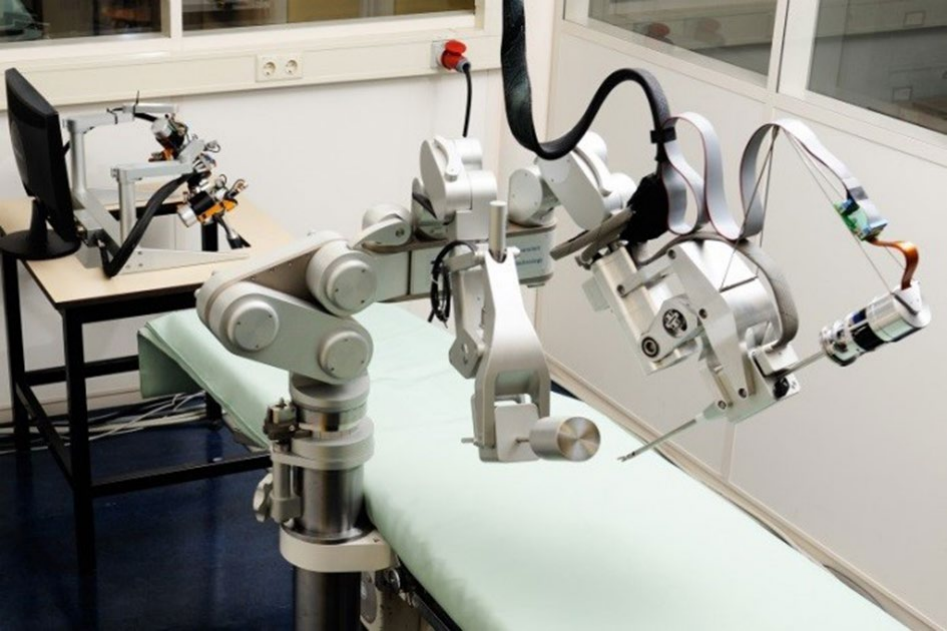
Surgical robot “Sofie”. Reprinted with permission from [60]

An important feature of telerobotic systems for MIS is the use of haptic interfaces as implemented on a surgical robotic system called SOFIE (Surgeon’s Operating Force feedback Interface Eindhoven); it was developed to overcome certain limitations of minimally invasive surgery [60, 61] (Fig. 7). Characteristic features include the direct connection of the system to the operating table and the implementation of force feedback applied to the operator’s joysticks. SOFIE is based on a master–slave control architecture with the two components entirely separated at some distance from each other [62]. The slave is a robotic arm frame, which can house three independent manipulators (one for a camera and two for surgical tools).

**Fig. 8:**
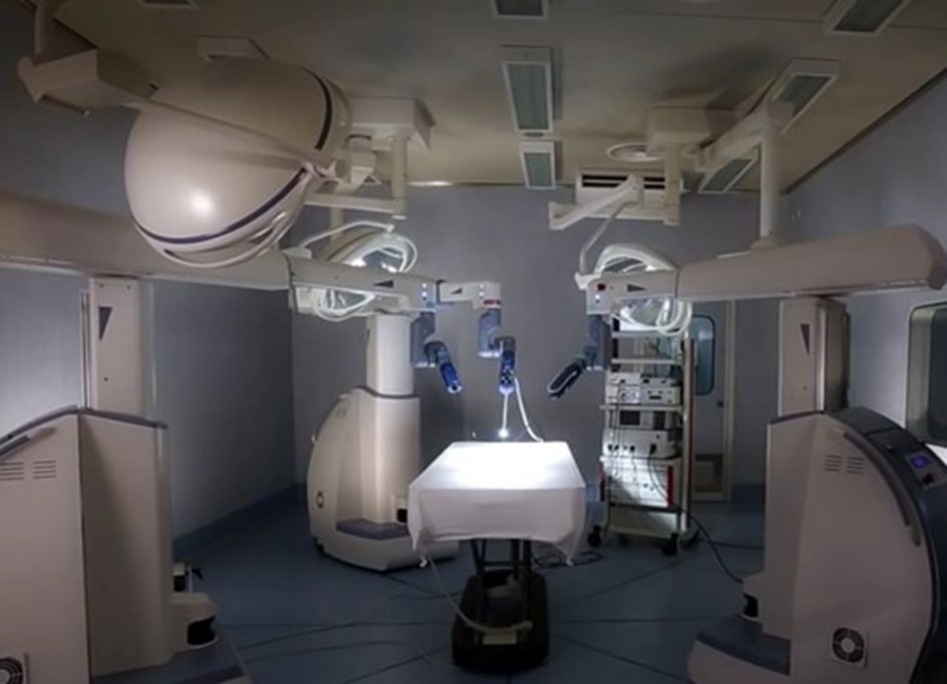
The Telelap ALF-X system consists of a console and three/four independent arms each one of which with six degrees-of-freedom. Reprinted with permission from [63]

A different system that also involves multiple arms was named Telelap ALF-X [63, 64] and is shown in Fig. 8. It is a surgical telerobotic system consisting of four arms with 6 DOF each. It facilitates remotely-operated 3D endoscopy procedures by utilizing haptic sensation, an eye-tracking system and configuration versatility. It consists of a remote control unit with haptic controls, the manipulator arms, and reusable endoscopic instruments. All instruments are connected to the arms with magnets for quick exchange of instruments. In telerobotic systems with multiple arms it is common that one of them is dedicated to holding and manipulating the laparoscopic camera. Such a setting is also found on the Al-Zahrawi system [65], which is composed of two separate parts, the master console and the slave system. They interact via an RS485 interface communication link. The master console includes the master manipulators, visual information presentation apparatus, and foot pedals. The slave station is composed of three manipulators (each one endowed with 6 DOF). Two of the manipulators are equipped with surgical instruments, whereas the one in the middle holds the endoscope. The manipulators utilize a modified double parallelogram mechanism to implement a remote center-of-motion approach.

In various MIS applications, it is useful for the robot to have many DOF. The dexterity afforded by constant curvature (flexible) or snake-like robot configurations is often desirable as in the case of the robot presented in [66]. The specific system is designed for MIS of the throat and upper airway. The slave system includes three snake-like arms with 5 DOF each and a laryngoscope. These robots are highly dexterous, as required, for surgical tool manipulation and suturing in confined spaces.

In cases of MIS procedures, where the number of incisions to access the internal anatomy is only one, the procedure is referred to as Single Port Access (SPA) surgery. SPA surgery can be facilitated by specially-designed continuous curving or snake-like robot systems and it was exploited in telerobotics as a branch of MIS. An example of such system is the insertable robotic effector platform (IREP) that was developed by Xu et al. [67] IREP can be deployed into a body cavity via a 15 mm diameter skin incision. It consists of two snake-like robots operating as slave surgical assistants for tissue manipulation, two parallelogram mechanisms with 2 DOF for the robots’ placement, and one controllable stereo vision module with two CCD cameras for depth perception and tool tracking. Each snake-like robot includes four components: (1) a gripper, (2) a 1 DOF wrist, (3) a 4 DOF snake arm, and (4) a flexible stem. It acts as a surgical telemanipulation slave for dual arm interventions and delivery of sensors or energy sources.

A different SPA surgery system was documented in [68]. The developed robot consists of a positioning manipulator (4 DOF), and a sheath manipulator (2 DOF) with a snake-like structure. The positioning manipulator remains outside of the patient’s body and it uses the available six DOF to control the position and orientation of the devices residing inside the body (two tissue manipulators; one for gripping with 5 DOF and the other for cautery with 3 DOF). The forefront of the sheath manipulator has a base supporting the endoscope and the tool manipulators. The operator controls the robot from a console in the operating room, using visual feedback supplied by the endoscopic video camera. The established wired communication (LAN) between the two PCs involved is based on the UDP protocol. Another example of master–slave teleoperated robotic system for MIS, called Single-Port lapaRoscopy bImaNual roboT (SPRINT), was developed by Petroni et al. [69]. SPRINT was designed for bimanual interventions through a single-access port. The key elements of the system are the master console, the insertion tube, the stereoscopic camera, and the slave robotic manipulators. Each robotic arm is a 6 DOF serial-chain miniature manipulator, while an additional 1 DOF is used by the end tool. The arms may be inserted into a cylindrical introducer that has a maximum diameter of 30 mm. The master console is composed of a 3D high-definition monitor, two hand controllers, and a foot-switch. The 3D display receives the images from the stereoscopic camera and, in combination with polarized glasses, provides depth perception and thus resulting in a fully immersive visualization system. Haptic interfaces are used at the master side for controlling all 6 DOF of each robotic arm, while two custom finger levers provide the control of the graspers.

A special case of MIS is the NOTES. Natural body orifices (e.g., mouth, nostrils, vagina, urethra, and rectum) are used in order to provide surgical instruments with an entry point to access internal organs (e.g., stomach, bladder). A teleoperated endoscopic system for NOTES procedures was proposed in [70]. It consists of two flexible hollow arms which are attached to a conventional flexible endoscope with 2 DOF to be used for visual feedback purposes. Surgical instruments are inserted through the arms to reach the operating area. The orientation of the endoscope and the arms is cable-driven using motors. The surgeon master console carries the appropriate interfaces and monitors for displaying the endoscopic images and other visual information. Another system dedicated to NOTES applications [71] comprises of a miniature robot with two arms and a main body. The robot can be advanced through the esophagus and into the peritoneal cavity using an overtube and an endoscope. After the robot is entirely inserted into the body, it provides a steady platform for visualization and dexterous manipulation. In vivo testing of the system using a porcine model demonstrated that the surgeon was able to explore the abdominal cavity and perform small bowel dissection.

A remote-controlled master–slave robot for vascular interventions (RVIR) was presented in [72]. The master site is located in an isolated cabinet with a protective lead glass window. The medical robot consists of a supporting manipulator and a catheter navigator is attached to it. The former can be positioned to an arbitrary posture. The slave site is located in the operating room and it also includes a rotational C-arm Digital Subtraction Angiography (DSA) imaging device. The remote control consists of a 3 DOF haptic device, allowing the doctor to sense the force between the catheter tip and the blood vessel wall.

Special-purpose telerobotic systems aim at performing specific operations more effectively. Such is the case of the telerobotic-assisted drilling system proposed in [73] that targets oral surgery and orthopaedics. The control is based on a master–slave architecture and it incorporates both position and force scaling options. The estimated cutting torque and force are graphically displayed on the monitor in real time, so that the surgeon has a perception of the applied force.

**Fig. 9:**
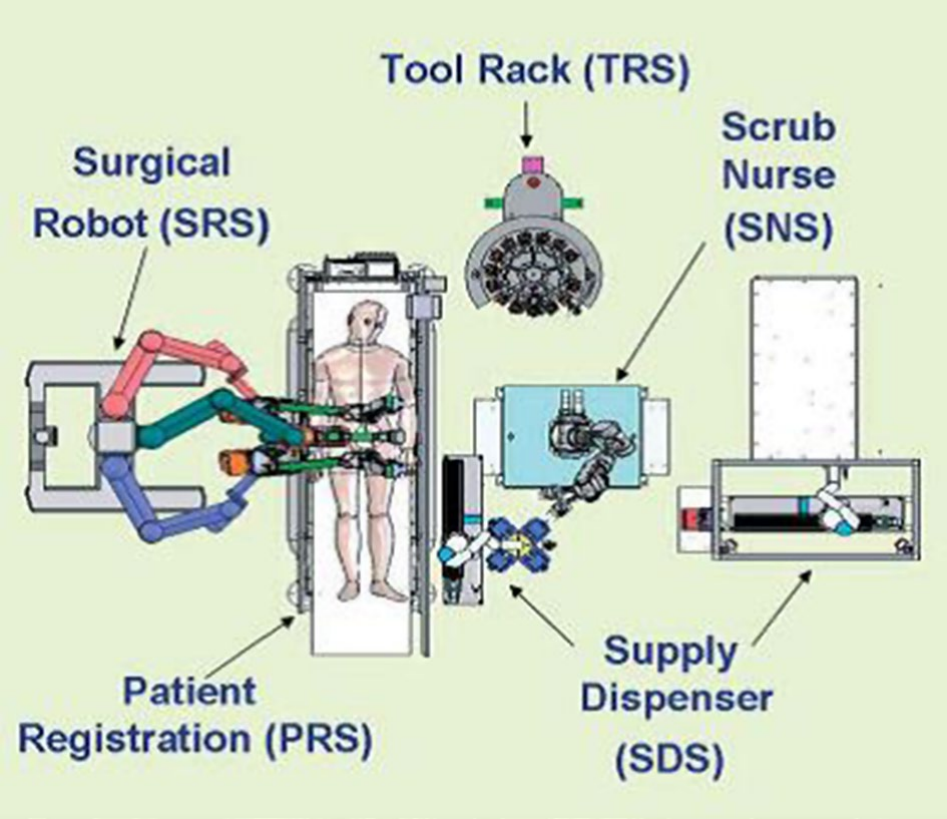
Layout of Trauma Pod system main components. Reprinted with permission from [74]

In order to remedy situations where a qualified scrub nurse is not present at the patient’s site, Garcia et al. [74] developed a semi-automated telerobotic surgical system that is capable of performing stabilization procedures with the patient being the only human in the surgical cell. The overall system, called the Trauma Pot, is based on the integration of several individual medical robotic systems into a unified mobile system, for battlefield use, as shown in Fig. 9. The latter consists of a da Vinci telesurgical robot, a scrub nurse robot, a supply dispensing subsystem, and an automatic tool rack. The proposed robot has three arms under the surgeon’s control. One is dedicated to holding the endoscopic camera and the remaining two for surgical tools manipulation. It has been demonstrated that automatic tool change and supply delivery can be performed much faster when compared to the same procedures performed manually by nurses. The system also includes a tomographic X-ray facility for patient diagnosis as well as 2D fluoroscopic data to support interventions. In general, autonomous teleoperated surgical systems eliminating the need of a scrub nurse are particularly important. This was also the objective of Ki-Young et al. [75] who developed a slave manipulator with roll-pitch-roll wrist and automatic tool loading and unloading mechanism for telerobotic surgery. In this system, the height of the slave manipulator can be adjusted by means of a vertical lifter while the horizontal position is manually adjusted. The slave manipulator holds an interchangeable surgical tool unit introduced into the abdomen and is designed to have 6-DOF plus a gripping DOF.

### Eye surgery and ENT surgery

It is envisioned that telerobotic technologies will play a key role in ophthalmological interventions, despite their relatively limited presence in the current literature. Ophthalmological interventions necessitate accurate and delicate motions which can be provided by robotic systems. One example toward this direction, is a robotic system for microsurgical keratoplasty which was developed by Hu et al. [76]. The system includes a microsurgical robot and an interchangeable end-effector, allowing surgical trephination and suturing under visual servoing control. The surgical information, such as puncture force and cutting depth, can be measured and processed in real time. An interactive user interface facilitates the robot control and planning of the robot-assisted procedure. A vision module is used to obtain images using two video cameras attached on the surgical microscope.

**Fig. 10:**
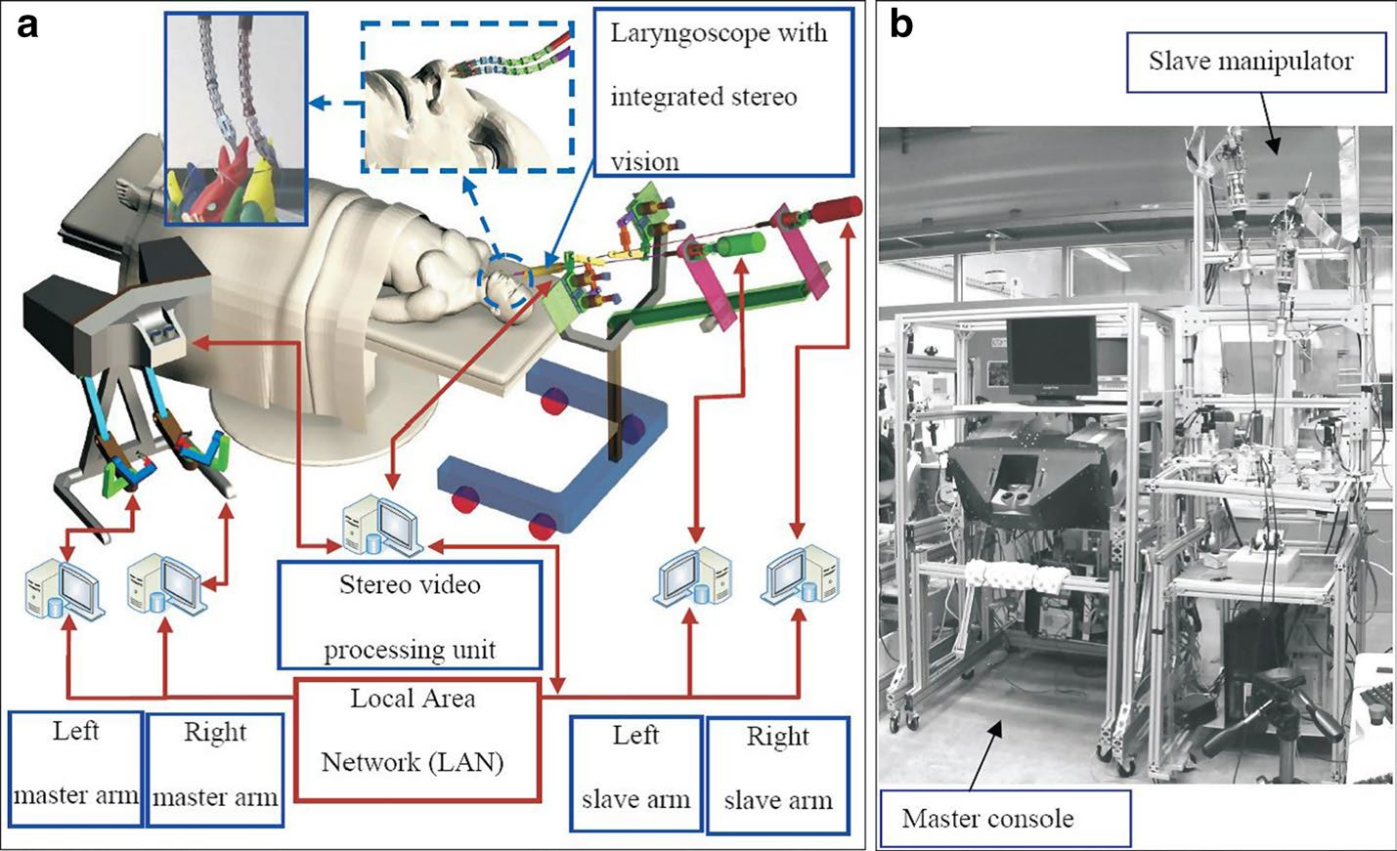
**a** Outline of the telerobotic system for MIS of the throat and upper airways. **b** The system prototype. Reprinted with permission from [66]

Special applications considered for teleoperated robots also include ear, nose, throat (ENT) surgery. A representative example is the system developed by Simaan et al. [66] dedicated to minimally invasive surgery of the throat and upper airways (Fig. 10). The system includes a dual-arm telesurgical slave with a total of 20 joint-space DOF. Each arm includes a distal dexterity unit which is a snake-like segment that allows for detailed and accurate dexterous operations in confined and limited spaces.

A telerobotic system was used by Dharamsi et al. [77] to examine the feasibility of a rapidly deployable telerobotic system for enabling transnasal microsurgery of the larynx and upper airways. The system comprises a continuous curving robot with 5 DOF with instrumentation ports through which can pass the fiber optic endoscope and a flexible needle. The actuation unit was installed with force sensors associated with all controlled axes. Studies using a human intubation trainer mannequin as well as cadaveric studies demonstrated that the system can be inserted transnasally and the needle with minimal forces on the surrounding tissues. Predetermined points were targeted in order to simulate an injection larygnoplasty procedure.

### Neurosurgery

Robotic features such as positioning accuracy and the steady-hand characteristic have driven developments in neurosurgery robotics, a branch of telerobotic systems that has received significant research attention over the past decade [78, 79]. A telerobotic master–slave system for minimally invasive neurosurgery, called LANS (Linear Actuator for Neurosurgery) was presented in [80, 81]. The slave station is designed so as to linearly move a tool (e.g., laser pointer, biopsy needle, low-energy X-ray emitter) along a pre-planned axis. The tool insertion into the brain is then guided by the surgeon through the haptic master incorporating force feedback. Experimental investigation concluded that the high accuracy of the system linked with the implementation of a scalable force feedback mechanism and a programmable virtual environment, can significantly decrease the invasiveness and improve the result of stereotactic neurosurgical actions, which are typically performed using stereotactic head frames and manual tool insertion instruments.

An image-guided, MR-compatible, computer-assisted robotic device for neurosurgical applications, termed ‘NeuroArm’, was developed in [82, 83]. The system delivers the sound, sight, and touch of surgery to an operator located at a remote workstation. Manipulation is based on a telerobotic system for microsurgery and stereotaxy and uses a master–slave control architecture. The system combines two MR-compatible remote manipulators with 7 DOF each, attached on a mobile base. The end-effectors interface with microsurgical tools and they are equipped with 3D force sensors, rendering the sense of touch via the haptic controllers. The workstation reconstructs the sensation and the sight of microsurgery by presenting the surgical site and 3D MRI displays, with superimposed tools. NeuroArm is able to cut and handle soft tissue, dissect tissue planes, perform suture, biopsy, electrocauterize, aspirate, and irrigate.

Among other MR-compatible systems for image-guided neurosurgery is a hydraulic/pneumatic-actuated telerobotic system proposed in [84]. The specific system is designed to perform neurosurgical procedures inside a closed-bore 1.5 Tesla MRI scanner. Potential applications include thermal ablation, radio frequency ablation, deep brain stimulation, and targeted drug delivery. A different robotic platform for micro-neurosurgery based on the master–slave paradigm was developed by Mitsubishi et al. [85]. A position–orientation decoupled design is employed to enhance positioning accuracy. More specifically, the master manipulators compute the surgeon’s motion which is precisely reproduced by the slave manipulators. Motion scaling is one of the available features. The platform further incorporates a high-definition stereomicroscope, which allows surgeons to move robotic forceps with 3D perception. The master and slave systems have dedicated real-time controllers which communicate using the User Datagram Protocol (UDP). The master and slave units are located in the same room and therefore the delay in communication is negligible. However, given the fact that the proposed system can be potentially used in a long-distance telerobotic scenario, end-to-end delay may become a critical issue.

### Cardiac and thoracic surgery

The fine and accurate motions that robotic manipulators can produce make telerobotic systems well-suited to cardiac and thoracic surgery [86]. Mayer et al., presented a telemanipulator for robotic heart surgery in [87]. The system consists of two robotic arms with 8 DOF each. The used minimally invasive instruments are equipped with strain-gauge force sensors that can measure forces along the three translational directions of the instrument. Forces are displayed to the user via two haptic devices and guidance is based on images from an endoscopic stereo camera. In order to emulate a stereoscopic impression, images can be displayed by means of either a head-mounted display, a cathode ray tube (CRT) screen equipped with shutter technology, or a 3D video projection.

A master–slave robotic system for MIS was presented in [88]. The system is part of an integrated telepresence environment for MIS [89]. Its purpose is to enable surgeons to perform operations requiring a high degree of manipulability such as minimally invasive coronary artery bypass operations on the beating heart. The slave system consists of three surgical arms, two of which carry actuated and sensor-integrated surgical instruments. The master console enables the surgeon to control the instruments utilizing the available stereo images of the operation site. Integrated haptic hand controllers register the hand movements, and also display the manipulation forces and torques. In addition to the master–slave operation mode, the system also facilitates a semi-automatic mode, where part of the procedure is performed autonomously by the robot.

A system that addresses kidney tissue ablation by high-intensity focused ultrasound (HIFU) was developed by Hacker et al. [90]. The robot-assisted treatments are performed using single or multiple probes. As stated by the authors, nephron-sparing surgery is an alternative method to radical nephrectomy for the treatment of renal tumours smaller than 4 cm. The objective of the proposed system is the reduction of the invasiveness of renal surgery through the use of percutaneous procedures and techniques, and tissue ablation methods such as radio frequency (RF) ablation, cryoablation, and HIFU.

A spherical wrist mechanism robot aiming at replacing humans assisting in laparoscopic surgeries, by manipulating the laparoscope during lengthy operations was proposed by Hsu et al. [91]. In addition, an automatic tracking and gesture recognizing subsystem was also developed, in order to provide a fully surgeon-based automatic integrated system, by tracking the tip of a surgical tool and moving the laparoscope-holding robot so as to keep the tool within the camera view. A robotic forceps manipulator for laparoscopic surgery using the double-screw-drive (DSD) mechanism was presented by Ishii et al. [92]. Its gripper can rotate so that it can provide enhanced maneuverability in laparoscopic surgery. In order to manipulate the developed multi DOF robotic forceps manipulator as a master–slave manipulator system, a joystick-type master manipulator and a servo system were developed.

Researchers in Canada [93] developed an experimental test-bed for robot-assisted image-guided minimally-invasive lung brachytherapy. The system combines a ZEUS surgical system (Computer Motion Inc.) with two arms for manipulating instruments and the AESOP (Computer Motion Inc.) robotic endoscope holder. Automated endoscopic system for optimal positioning (AESOP), is a voice-activated robot used to hold the endoscope. The instrument arms mimic the motion of hand-held instruments, which are manipulated by the surgeon from the remote console. One of the arms can be used to hold and manipulate the ultrasound probe, while the other holds the seed injector [93]. ZEUS was discontinued in 2003, following the merge of Computer Motion with Intuitive Surgical [94]. The new company instead developed the da Vinci surgical system. A demonstration of the Zeus system teleoperation capabilities was the assessment of the feasibility of laparoscopic robot-assisted pyeloplasty in a porcine model presented in [91]. The experimental setting involved satellite communications using the internet protocol virtual private network (IP-VPNe) demonstrating its potential for long-distance teleoperation. The da Vinci system itself, which was described earlier in “Short-distance paradigm: the da Vinci system” section, plays a key role in thoracic surgery.

A unique concept to cardiac interventions is an epicardial crawling robot for myocardial injections called Heartlander, which was presented in [95]. HeartLander is a miniature robot with the ability to “stick” to the epicardium, travel to the operative site, and perform intramyocardial injections under the direct supervision of a surgeon, which prevents further myocardial infarctions. The miniature robot adheres to the epicardium using suction supplied to its suction pads through vacuum lines in the tethering tube and the activation for movement is provided by three nitinol wires that pass through the tether and are driven by motor belts in the supporting instrumentation. Testing of HeartLander included in vivo studies on a porcine model [96].

**Fig. 11:**
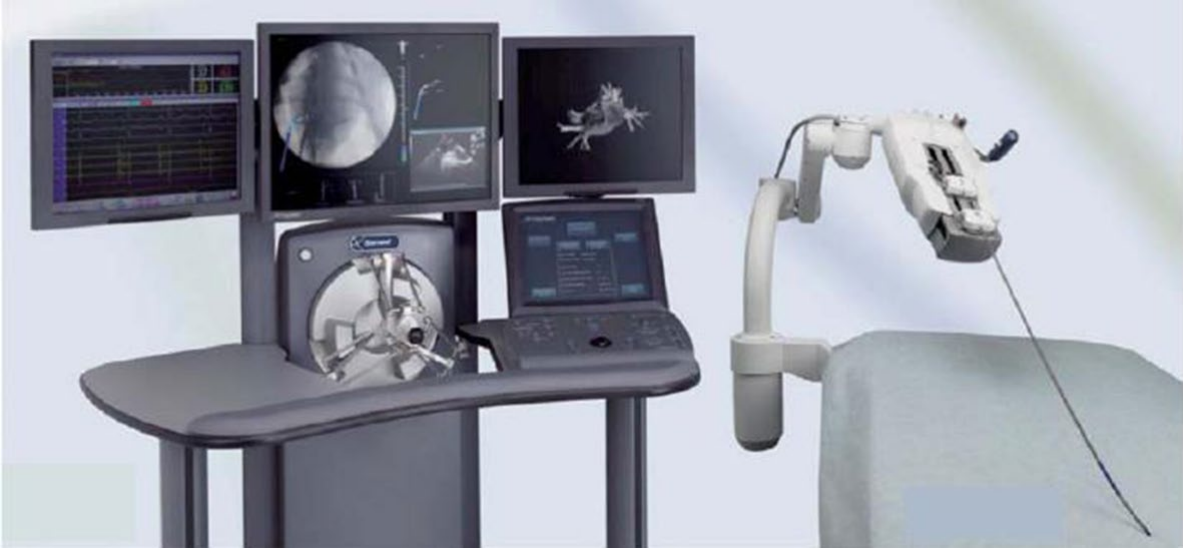
The Hansen robotic system includes the physician workstation and the remote catheter manipulator. Reprinted with permission from [98]

Cardiac systems also include a robotic catheter system called Sensei (Fig. 11) which was developed by Hansen Medical [97]. It was designed to operate in conjunction with the Artisan control, which consists of a steerable guide catheter and sheath. This contains a through lumen to accommodate the percutaneous catheters. It is reportedly the world’s first robotic transvascular aneurysm repair system. It translates surgeon’s hand motions, which are monitored by means of a commercial haptic feedback manipulator, into catheter motions inside the patient heart, thus facilitating accurate access to hard-to-reach cardiac anatomy. The Artisan catheter can be controlled in three dimensions exploiting the available 6 DOF and force-sensing capabilities [97, 98]. Lastly, we refer to the work of Castro et al. [99] who developed a Miniature Anchored Robotic Videoscope for Expedited Laparoscopy (MARVEL) and a Camera Module (CM) that features wireless communications and control. The MARVEL System consists of a wireless human machine interface (HMI) and a wireless laparoscopic CM attached to the abdominal wall. The surgeon interacts with the system through a standard joystick and a software control application.

### Gastrointestinal and colorectal surgery/examinations

Gastrointestinal and colorectal surgery requires dexterous multi DOF robotic systems. Biologically-inspired snake-like or other constant curvature robots are often well-suited for this application. A miniature robot for intestinal inspection based on the bio-mimetics of an earthworm was developed by Zuo and colleagues [100]. Its diameter and length are 7.5 and 120 mm, respectively, and the robot is driven using a direct current motor. Control is based on dedicated end-to-end serial link interface. The authors presented the structure and the locomotion mechanism of the robot whereas initial experiments using a robot’s prototype demonstrated its ability to navigate inside horizontal and inclined tubes.

A method followed by Ciuti et al. [101] is based on robotic magnetic steering and locomotion of a capsule endoscope for diagnostic and surgical endoluminal procedures. The system is composed of a human machine interface, a 6 DOF robotic arm capable of moving an external permanent magnet, and a capsular device, equipped with inertial and wireless vision sensors. The user interface displays the real-time images coming wirelessly from the capsular device together with other information related to the state of the platform. Another approach to capsular endoscopy that takes advantage of active legged locomotion in the gastro intestinal (GI) tract was proposed by Quirini et al. [102]. Compared to earlier capsule robots, this robot uses legged locomotion instead of just floating through the GI tract. On-board locomotion mechanisms allow the capsule to travel through the GI tract while avoiding bulky external driving systems as in the case of externally actuated devices (e.g., magnetically actuated systems). Two different prototypes were developed, having four and eight legs, respectively.

A micro-wireless robotic endoscope for human GI examinations was developed by Wang et al. [103]. The robot’s diameter and length was 10 mm and 190 mm, respectively. A locomotion principle based on bio-mimetic earthworm is adopted aiming at a higher adaptability to the GI tract. The robot was composed of three linear driving cells and energy is continuously supplied in real time by an energy-transmitting system based on electromagnetic coupling.

### Urologic surgery

Robotic systems are becoming increasingly important in urologic surgery [104]. The da Vinci system is one of the prevailing systems currently in use by surgeons worldwide for prostatectomy and other urologic procedures. A 4 DOF robot for prostate brachytherapy was developed by Salcudean et al. in [105]. The robot can translate a needle guide in the X–Y plane, allowing for precise needle insertion along the Z direction. It can also rotate the guide about the X and Y axes, providing fine control over the needle insertion point and orientation. The system can be mounted on a standard brachytherapy stepper.

Based on high-quality intraprocedural anatomical and functional images, MR image-guided procedures can be carried out with more precision and more effectively. Various systems were developed for this purpose. Goldenberg et al. [106] reported on the development of a closed-bore MR-compatible robotic system for image-guided prostatic interventions, namely ablation, brachytherapy, and biopsy. This type of robots use either ultrasonic direct drive motors for actuation to avoid metallic and solenoid parts, or (non-metallic) cables remote actuators The first stage of development addresses laser-based ablation. Fischer and coworkers [107] designed an MR-compatible robotic assistant system that can be used for needle placement in the prostate for biopsy and brachytherapy procedures. The robotic system was designed in such a way, so as to be able to operate in the limited space, between the patient’s legs within a leg rest/tunnel in a high-field, closed-bore MRI scanner.

### Spinal intervention

Robotic systems enable spine surgeons to perform complex spine surgeries improving their accuracy and safety [108, 109]. Telerobotic systems can be used in various cases including the treatment of degenerative spinal conditions, spine tumors, and spinal deformities. Ju et al. [110] presented the SpineNav, a robot for percutaneous vertebroplasty, which could insert needles autonomously or using a tele-operated mechanism with 5 DOF. The robot was designed to be used inside a CT scanner, and thus its mounting platform has a metal mask which can be easily segmented from the intra-operative images, to estimate the robot’s base position and orientation with respect to the patient, as required for registration purposes. Testing suggested a mean positioning error of less than 1 mm [108].

An assistive robot for the spinal fusion surgery with a dexterous end-effector was developed by Lee et al. [111]. It was named Cooperative Robotic Assistant (CoRA) and it is capable of high-speed drilling for cortical layer gimleting and tele-operated insertion of screws into the vertebrae. The end-effector is position-controlled using the 5 DOF of the robot. The system is a closed kinematic mechanism providing extra stability as required to resist the strong reaction forces developed during the surgery. The robot permits the doctor to directly control the position and orientation of the end-effector.

The European project Microneuro-endoscopy of Spinal Cord (MINOSC) led to the development of a robotic system for interventions of the spinal cord from within the sub-arachnoid space [112]. It provides the surgeon with direct vision of the structures (i.e., spinal cord, roots, and vessels) and the possibility of performing specific operations such as local electrostimulation of nerve roots. A feedback control system can steer the endoscope tip to avoid obstacles. Steering is executed by a 2 DOF cable-driven mechanism and three lateral hydraulic jets that stabilize the endoscope’s tip.

## Long distance telerobotic systems

This section is dedicated to the review of long-distance telerobotic systems in which the master and slave sites are geographically separated. The review covers systems from the following areas: general surgery, spinal intervention, and tele-echography. The reviewed systems are tabulated in Table 4. The main difference with short distance telerobotic system is the data link between patient site (slave robot) and the expert site (master station). This link cannot be considered like an end-to-end standard link. It must pass through different networks (WAN/internet, Satellite, ISDN or 3G/4G nets) with different protocols that involve problems of quantity/bandwidth, quality, delay, jitter, and lag. This is the major problem, and we must adapt and robustify the control of the robot to these media.

**Table 4:** Long-distance telerobotic systems

	Name	Mechanical design	Application area	Status	References
1	Raven robot	Spherical	General surgery	EXP	[113]
2	Lapabot	Serial	General surgery—MIS	EXP	[114]
3	Internet based cather manipulating system	Cartesian	General surgery	EXP	[115]
4	RIME	Serial	Spinal intervention	EXP	[116]
5	Robotized tele-echography MELODY	Serial	Tele-echography	COM	[49, 119]
6	Free hand controller for remote ultrasound imaging	Parallel	Tele-echography	EXP	[120]
7	Wearable tele-echograpgy robot for FAST	Pitching, rolling positioning	Tele-echography	EXP	[121]
8	Servo actuated robotic arm for tele-echography	Serial	Tele-echography	EXP	[122]
9	Parallel robot for ultrasound imaging	Parallel	Tele-echography	EXP	[117]

*EXP* experimental, *COM* commercial

### General surgery

The telerobotic surgical system called “RAVEN” was introduced in [113]. It includes three elements: the patient site, the surgeon site, and a network connecting the two. The patient site comprises of two surgical manipulators that are positioned over the patient by passive macro-positioning arms. The surgeon site consists of two PHANTOM Omni devices (SensAble Technologies, USA), a USB foot-pedal, a laptop running the surgeon’s graphical user interface software, and a video feed of the operative site. The communication can be established over any packet-based network such as a local private network, the Internet, or wireless network, and employs the UDP protocol for minimizing time delay. The incorporated 7 DOF cable-actuated surgical manipulator aims at providing motions similar to manual MIS as well as wrist joints located at the surgical end-effector.

**Fig. 12:**
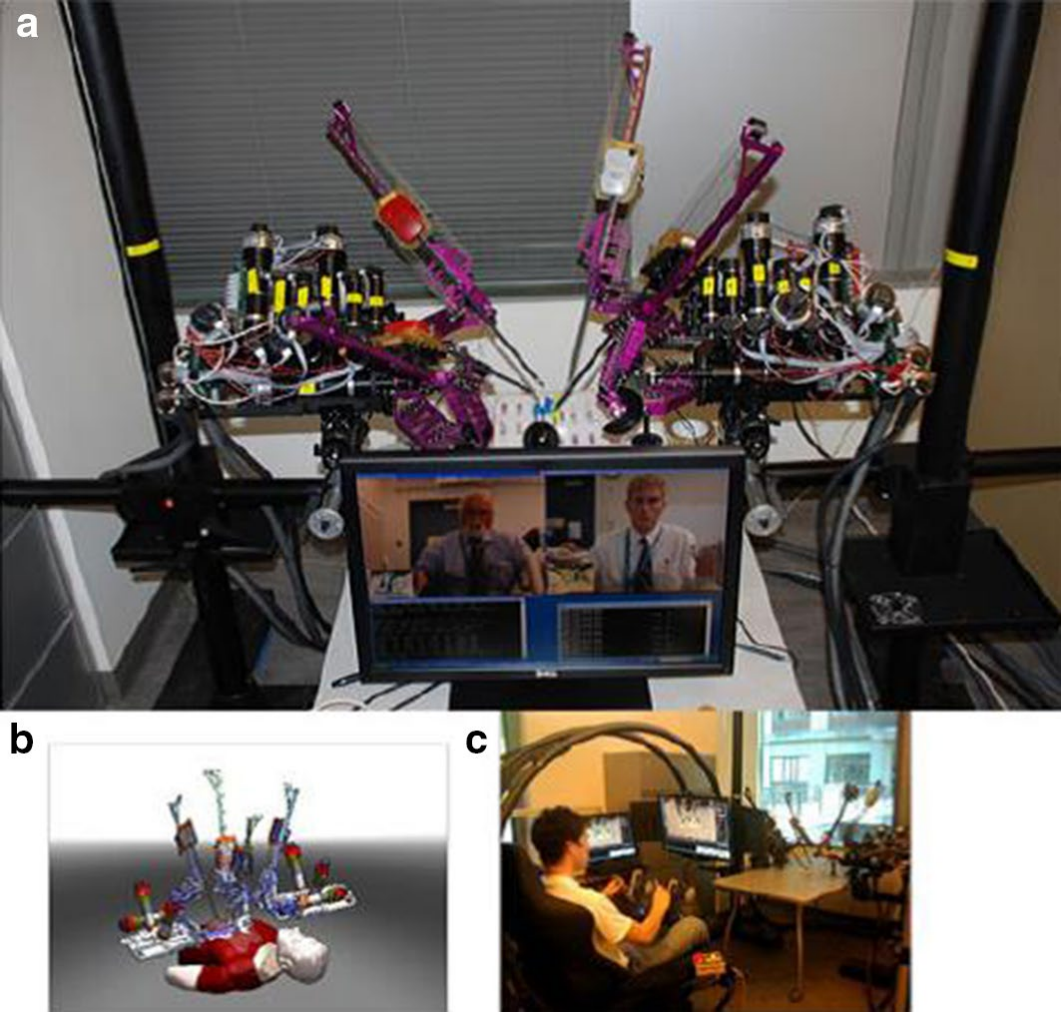
**a** Four Raven‐II robotic arms and two cameras arranged for collaborative telesurgery; **b** CAD rendering of the system; **c** Surgical console. Copyright © IEEE. All rights reserved. Reprinted with permission from [116]

An advantage of the RAVEN system is that it only weighs approximately 22 kg. It was tested hundreds of feet underwater by the National Aeronautics and Space Administration (NASA) in order to examine the robot’s appropriateness for telerobotic surgery in space. The RAVEN system provides for direct teleoperation but does not accommodate any advanced computational functions (e.g., motion planning, machine learning, stereo vision, tactile/haptic feedback) [6]. RAVEN II (Fig. 12), is a newer version with 7 DOF, compact electronics, and two wing-like arms which end in tiny gripper claws, designed to perform surgery on simulated patients.

The laparoscopic surgical system developed by Choi et al. [114] features multiple compact slave manipulators. The system can simultaneously operate one laparoscope arm and up to four instrument arms. Moreover, it can be directly attached to the operating table. The slave robot is controlled remotely through a wired, packet-based ethernet network. The master console provides input and output terminals for the operator. The input terminals include left and right master handles, as well as five foot pedal switches for emergency stop, temporary halt/resume, and electrocautery output on/off. The output terminals include a touch-screen monitor to display and adjust the functional status of the controllers, and two video monitors for laparoscope display and external monitoring camera display. The master handle has a 5 DOF structure, and its joints correspond to the ones of the 5 DOF slave manipulator. The driving mechanisms are compact and conventional laparoscopic instruments are utilized without modification.

A catheter manipulation system developed by Guo et al. [115] is also based on a master–slave structure. The surgeon’s console is the master side and the catheter manipulator is the slave side of the system. The manipulator which is at the patient side has two DOF: one is the axial and the other is the radial movement along a supporting frame. An internet-based communication between the controller and the catheter manipulator was employed, while a server-client structure realizes the communication. Two kinds of data are transmitted between the server and the client. One is the control data between the master and slave stations such as handle rotations and movement stage displacements. At the same time, rotation of the catheter, displacement of the movement stage, force data from a load cell, and torque data from a torque sensor are sent to the master side. The second type of data is image data acquired and transmitted by an IP camera. Compared with control data, the amount of image data is considerably higher. To maintain the integrity of the operation and ensure the appropriate rate of the data flows, these two types of data are transmitted separately. Testing involved two-way remote control experiments carried out between China and Japan.

### Spinal intervention

**Fig. 13:**
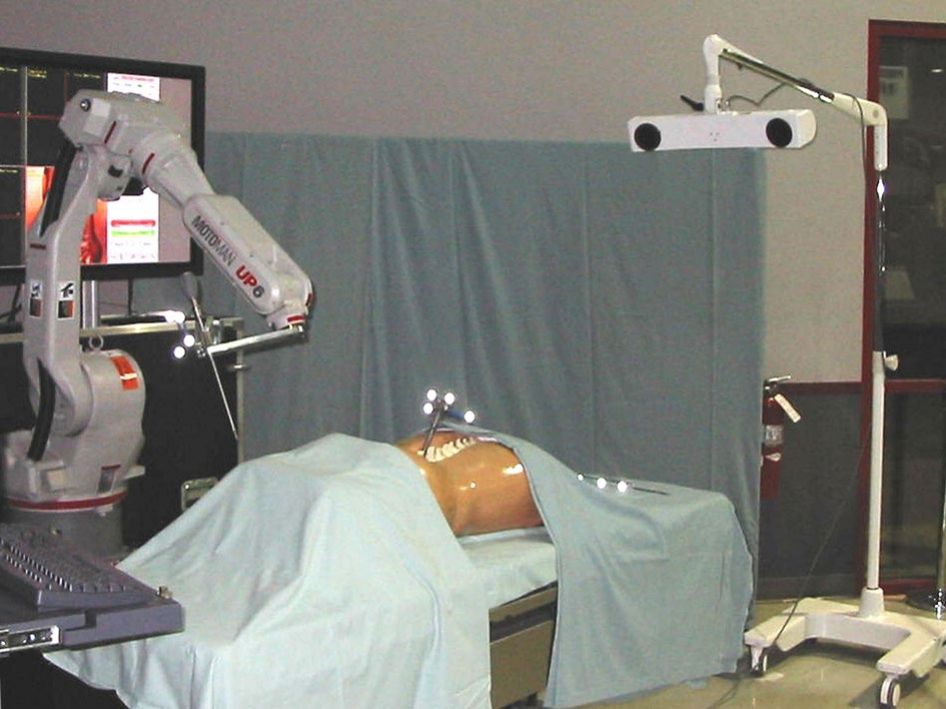
The RIME surgical robotic system developed by Wright State University. Copyright © IEEE. All rights reserved. Reprinted with permission from [142]

As part of the project Robot in Medical Environment (RIME), Boschetti et al. [116] proposed a robotic system for drilling in transpedicular fixation surgeries. The project’s main contributions were the development of a fully teleoperated system, which allowed the surgeon to operate on a patient who could be kilometers away. The system, shown in Fig. 13, comprises a haptic master, a 3D visual feedback device, a slave robot, and a haptic server through which all the modules communicate. The use of the haptic server allows decoupling direct communication among all the devices involved in teleoperation and facilitates the possibility of introducing virtual forces at the master side, thus providing a considerable enhancement of operators’ performances. An internet-based communication between the haptic master and the slave robot is established and both sites use the UDP protocol for the exchange of data. Experiments reported by Rosati et al. [116] demonstrated the feasibility of haptic feedback transmission and control of the involved 6 DOF industrial robot between two cities separated by 35 km.

### Tele-echography

Various systems have been developed for robotic ultrasonography as described in [117] and the references therein. A prototype, portable robot named MELODY was developed based on the earlier Teresa system [49]. MELODY was manufactured and commercialized by AdEchotech Cie (France). This tele-echography system is based on a teleoperation scheme and consists of an electric motorized support holding the ultrasound probe, which can be arbitrarily oriented. It effectively reproduces all the movements of the medical expert’s hand located at the expert station [118]. A force sensor, embedded in the robot end-effector, measures the contact force between the real probe and the patient’s skin and enables to limit this force to 20 Newton, for patient safety purposes. The communication link between expert and patient site requires a minimum bandwidth of 256 kbps. The network links the two sites to exchange robot control data, ultrasound images, haptic information, ambient images and audio instructions. The communication link can be any packet-based network and the system uses both TCP and UDP protocols. Additional information about the system can be found in “Long-distance paradigm: the MELODY system” section. The current version of the commercial system is named MELODY [119].

A hand controller, suitable for remote ultrasound diagnosis was developed by Farshid and Najafi [120]. It is built upon parallel mechanisms and has 4 DOF to provide standard clinical motions of ultrasound imaging. Its operation is based on a remote center-of-motion principle and it exhibits a one-to-one mapping between its movements and the movements of the ultrasound probe at the remote site. The prototyped hand-controller was used as a master device in a real remote ultrasound imaging task. The patient site included a robotic wrist. Both patient and physician sites were connected through a UDP communication network. After a very brief training a clinician successfully captured ultrasound images of a volunteer’s heart and kidney.

**Fig. 14:**
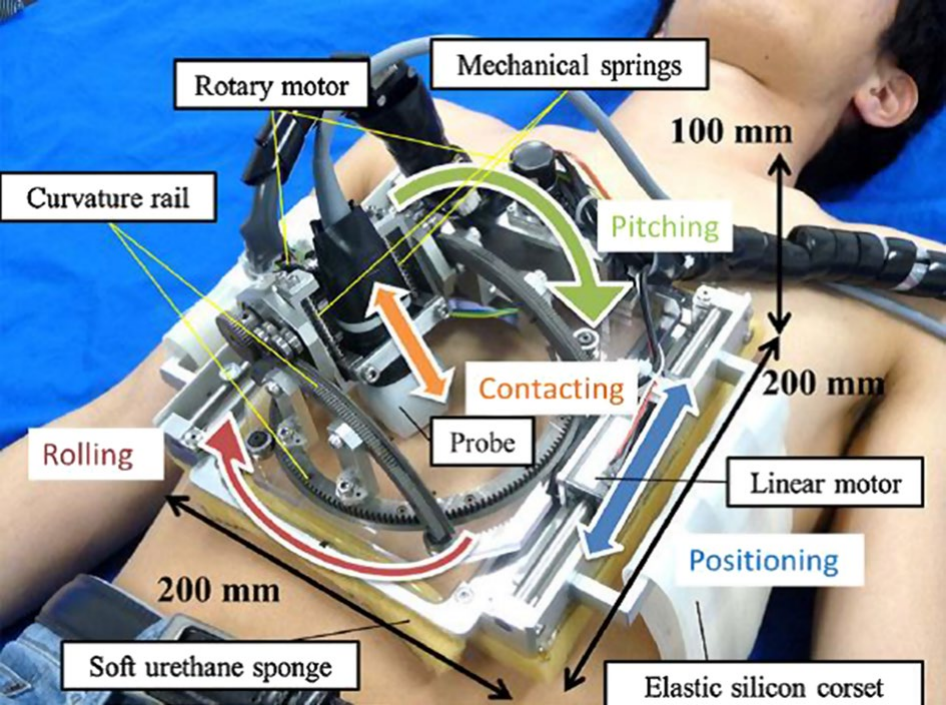
Wearable teleechography robot. The robot provides 4 DOF and control of the US probe for FAST. Reprinted with permission from [121]

A wearable tele-echography 4 DOF robot for Focused Assessment with Sonography for Trauma (FAST) was presented in [121] (Fig. 14). A medical expert uses a graphical user interface on the computer from a remote hospital, and the control signals are transmitted through a network to the robot in an ambulance or an injury site. Information concerning the position and orientation of the ultrasound probe, as well as image and voice information, are also transmitted. The medical expert performs the tele-echography while observing the echo image, the US probe, and the patient. The portable echo device consists of a MicroMaxx (SonoSite Inc.) and a sector US probe. A portable battery for the system is also required. The whole setup can function over different types of packet-based wired and wireless networks such as LAN, 3G, 4G, and mobile WiMAX, in order to control the wearable tele-echography robot and transmit audio and ambient data.

**Fig. 15:**
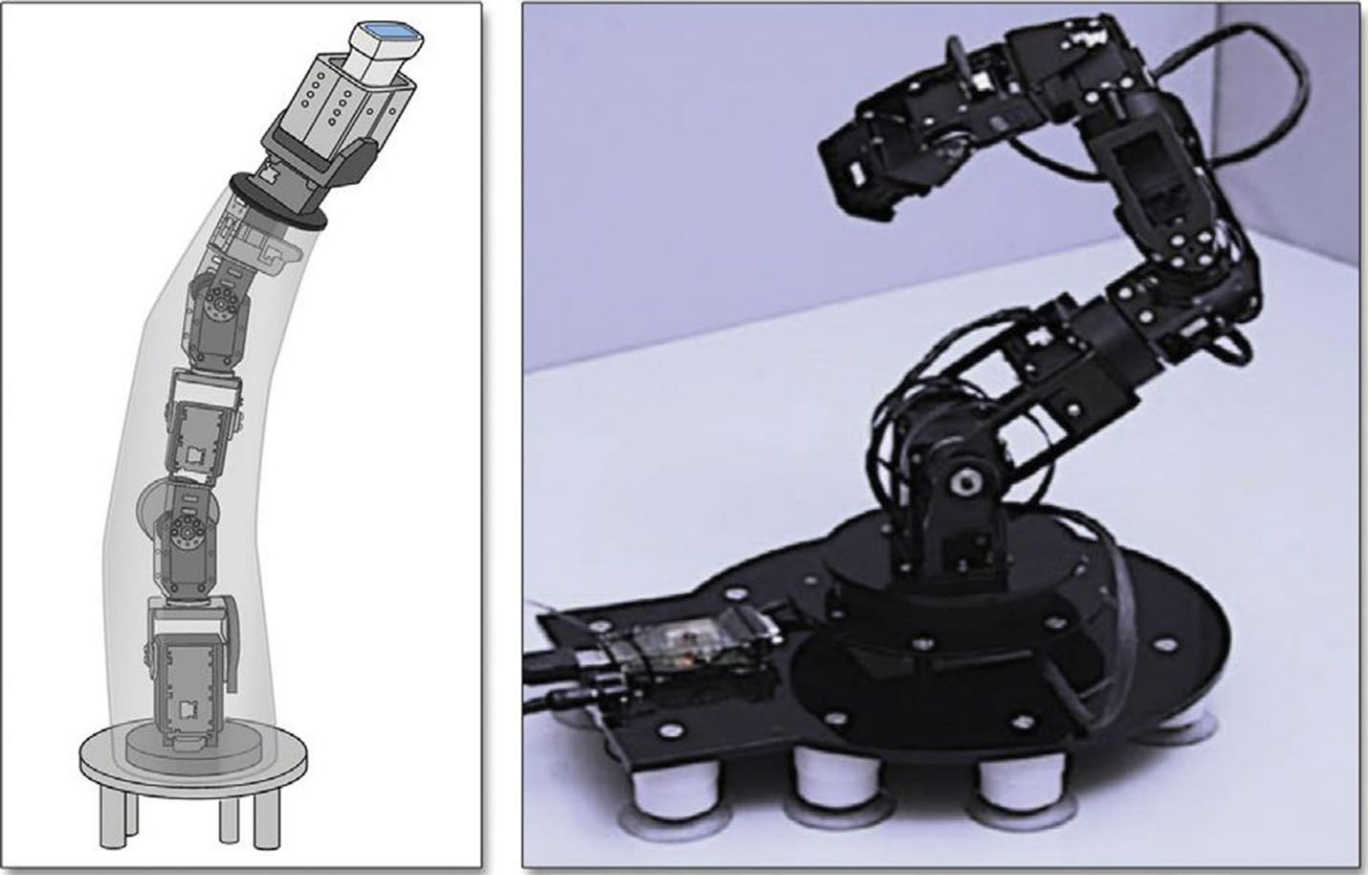
Robotic arm for slave station of telerobotic ultrasonography platform. Reprinted with permission from [122]

Telerobotic ultrasonography was also investigated by Sengupta et al. [122] using a customized lightweight robotic arm with 7 DOF (Fig. 15) and a specially designed end-effector on which the transducer is attached. In general, a high-bandwidth dedicated telecommunication line or a dedicated high-speed terrestrial fiber optic network was employed for this purpose. Intercity and Trans-Atlantic telerobotic ultrasound teleconsultations were performed from master stations located in New York, USA and Munich, Germany, and imaged a phantom and a human volunteer located at a slave station in Massachusetts, USA using broadband Internet of 100 and 50 Mbps, for the Intercity and Trans-Atlantic teleconsultations, respectively. Implementation was supported by video (using a dual camera system) and sound feed to allow the interaction between the operator and the subject while acquiring real-time ultrasound images. Control at the master station was based on a conventional mouse, the dials of the remote control interface, and the keyboard.

A 6-DOF parallel robot for telemanipulating an ultrasound imaging probe was presented by Monfaredi et al. [117]. Gross positioning of the robot can be realized through a passive or active manipulation system before the sonographer begins with the fine movements through the parallel robot. The parallel robot consists of three legs and two plates one of which is fixed. The moving plate hosts a force/torque sensor and the ultrasound probe. A phantom study was briefly reported where a general-purpose robotic manipulator was involved for the gross positioning the ultrasound robot. Video captured at the remote site was presented to the operator in real time while using a haptic device to manipulate the robot.

## Discussion—future challenges

**Fig. 16:**
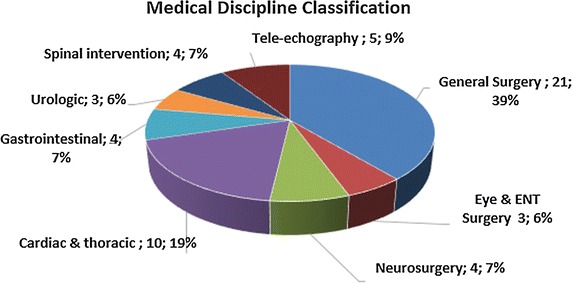
Medical discipline classification of the reviewed tele-robotic systems

**Fig. 17:**
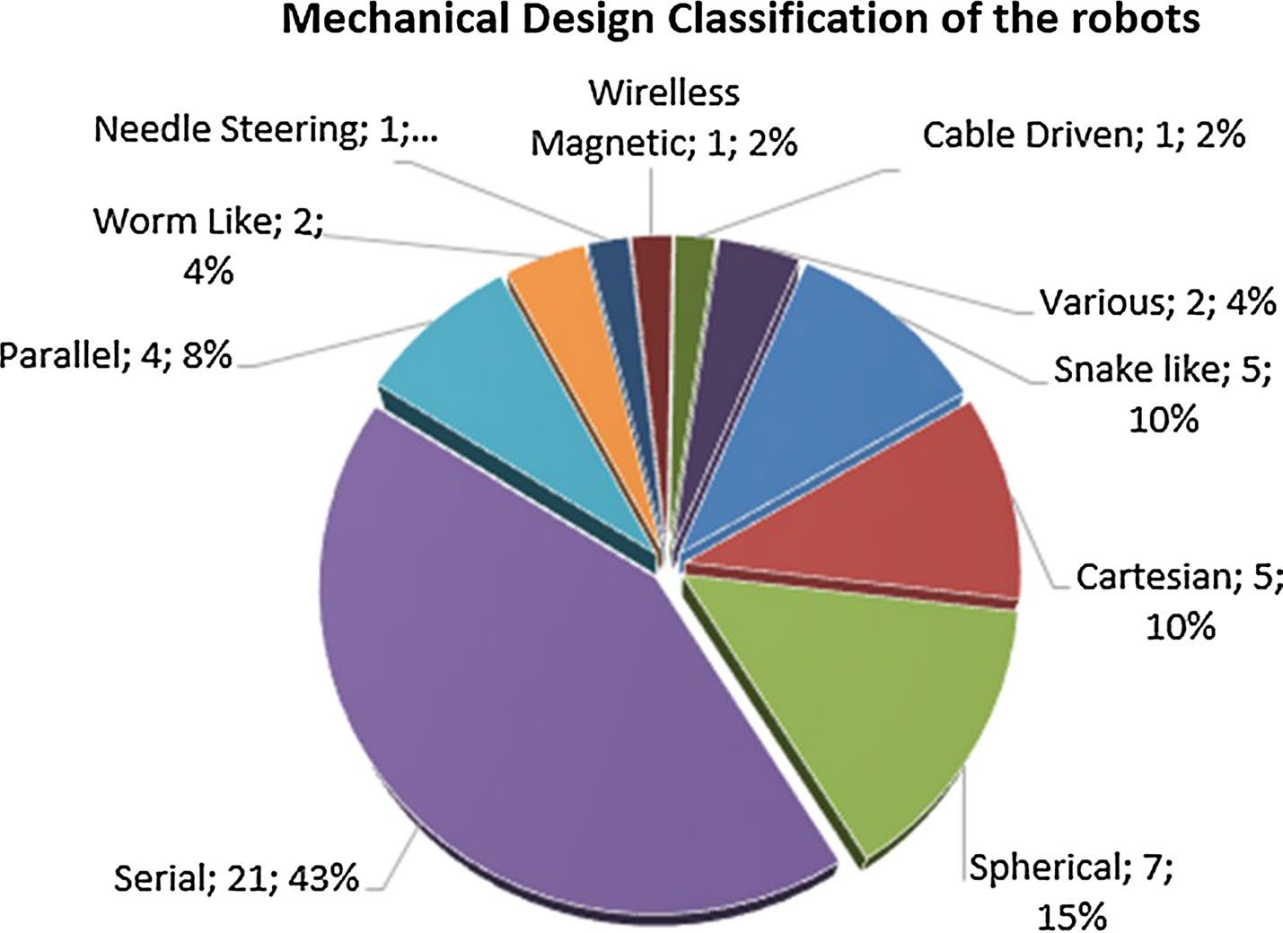
Mechanical design of the reviewed robotic systems

**Fig. 18:**
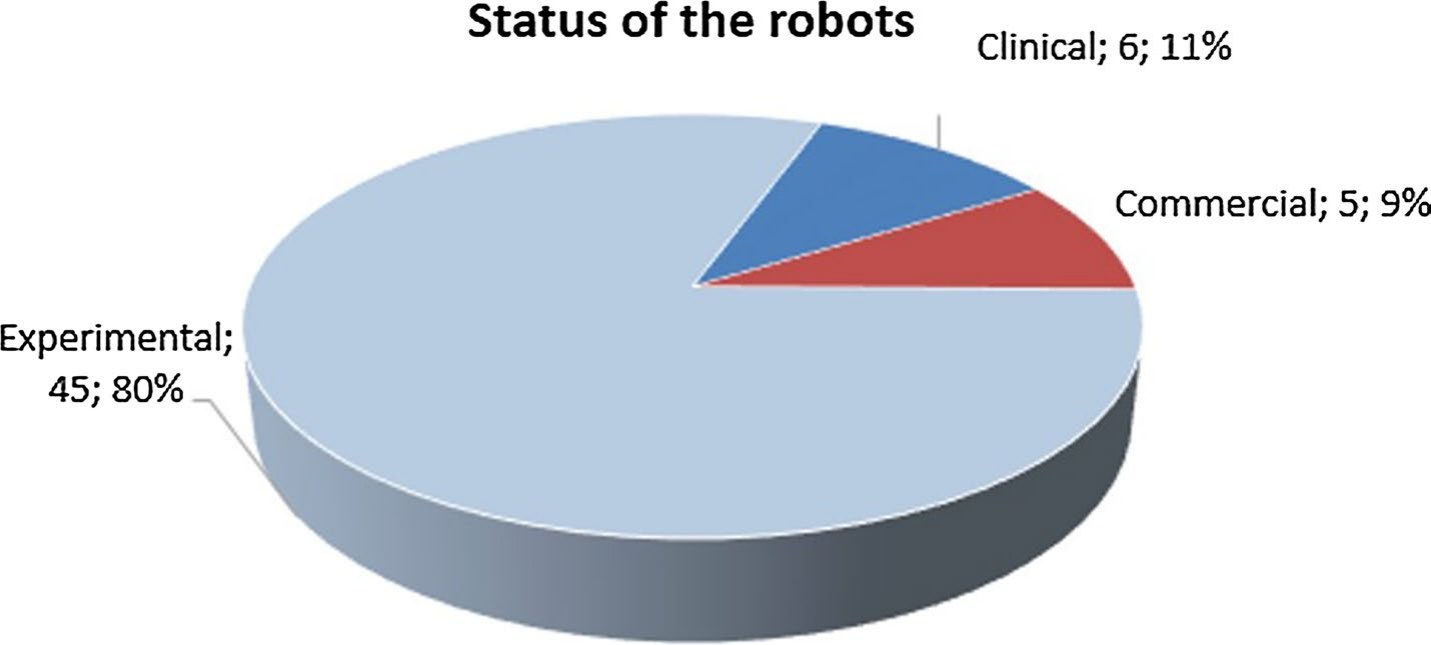
Status of the reviewed robotic systems

The present review study of medical telerobotics discusses the current trends, the potential applications and associated benefits, and highlights the future challenges as summarized in Table 5. General information about the considered telerobotic systems was consolidated earlier in Tables 3 and 4, as well as in the form of summary plots (Figs. 16, 17, 18) to highlight some key issues. Telerobotics have already been employed for a wide range of diagnostic and interventional applications in different medical disciplines, as depicted in Fig. 16, even though many general-purpose systems have been developed the majority of telerobotic systems are application/anatomy specific. Moreover, robotic systems used for telerobotic applications are diverse in terms of kinematic structure, degrees-of-freedom, and actuation methods (Fig. 17). Serial articulated robots have been considered in telerobotic applications but also other forms, including parallel robots and snake-like ones. Despite remarkable achievements demonstrated by a plethora of the examined telerobotic systems, yet only a handful of them have reached a commercialization stage, and even fewer have been adopted in clinical practice (Fig. 18). This fact signifies that further efforts are required to address both clinical and technological challenges.

**Table 5:** Challenges and areas for future developments in medical telerobotics

1	Regulatory approvals	Approvals take a significant amount of the development time and cost. Lack of worldwide acceptable regulatory standards makes the clearance process inefficient and costly
2	Clinical acceptance	Acceptance by clinicians and patients is required but also by third-party payers in the health-care system including insurance companies
3	Cost of acquisition and maintenance of telerobotic systems	These are mainly attributed to the high development costs related to the strict safety and reliability requirements
4	Interdisciplinary development approach	The development of telerobotic systems requires an interdisciplinary approach to deal effectively with both clinical and engineering aspects
5	Human factors	Human factors considerations need to be an integral part of the design to yield safer, more usable and effective devices. Decreased interaction among the healthcare professionals and patients during application needs attention
6	Telepresence enhancement	Available means include the development of effective user interfaces and use of force feedback haptic systems
7	Software tools	Emphasis required on preoperative planning tools. They may analyze imaging information, present the operator with optimal courses of action, and facilitate decision making
8	Radiological imaging methods	Apart from camera systems other imaging methods can be further exploited for visualization and guidance (e.g., US, CT, MRI)
9	Information fusing	Fusing intra-operative images with 3D patient-specific models constructed from pre-operative information enhances perception. Also, merging intra-operative information acquired from different imaging modalities (e.g., MRI and ultrasound) may improve visualization
10	Telecommunication networks	Long-distance telerobotics demand reliable transmission of huge amounts of data with acceptable delay. Latest technologies need to be embraced
11	Video compression technologies	Compression technologies will facilitate the transfer of large quantities of information
12	Network security enhancements	Wireless networks’ security vulnerability remains a major concern for the exploitation of (long-distance) telerobotics in telemedicine
13	Moral and legal issues	Transmission of information over communication networks raises issues regarding the protection of patient’s privacy and needs to be regulated. Legal regulation regarding application of medical telerobotics is also needed to prevent unauthorized service providers
14	Liability issues	Liability and responsibility for complications during a telerobotic procedure is among delicate issues to be formally addressed
15	Development of robotic comanipulation systems	Robotic comanipulation systems with required dexterity are needed while satisfying safety requirements
16	Robot control	The establishment of stable/robust control systems despite the long-distance data transmission involved presents engineering challenges
17	Auxiliary control functions	Implementation of auxiliary control functions will provide enhancements to long-distance telerobotics and reduce burden on the operating physician. Particularly important will be the biomotion compensation
18	Physicians training	The availability of trained physicians will require medical schools to acquire telerobotic technologies and introduce them in their educational programs. Development of training simulators will also play an important role in that respect
19	Telementoring and collaborative surgery	The telementoring capabilities of telerobotics can be further exploited to train and support physicians. Experienced physicians can play the preceptor’s role to other physicians without having to relocate
20	Collaborative research approach	Shared efforts between universities and companies will foster the development of new commercializable technologies

An existing difficulty for the introduction of medical robotic technologies remains the inertia of ongoing medical practice [3], which requires particular effort to overcome. A prerequisite for commercialization is to obtain regulatory approvals, which usually takes a significant amount of the development time and cost. In the United States, medical devices are approved for commercial use by the Food and Drug Administration (FDA) [123], and they also have to comply with the Quality System Regulation (QSR). For the latter, manufacturers are required to establish quality systems to ensure that their products consistently meet applicable requirements and specifications. The equivalent regulatory requirements for medical devices in Europe include the CE marking and also compliance with ISO 9001 and 9002 standards for the manufacturing processes. Even though in each case the requirements are similar, they are not identical, and products are often required to comply with both of them [124]. The importance of these standards is undisputable but an agreement on regulatory standards that are acceptable worldwide would facilitate progress by making the clearance process more efficient and cost effective. Another important issue regarding the commercial success of a medical device that goes beyond the regulatory approvals is discussed in [124]. For the device to be marketable, it must also be accepted by third-party payers in the health-care system, including insurance companies.

Another significant issue that limits the adoption of telerobotic—and robotics technology in general—in clinical practice is the high cost associated with the acquisition and maintenance of such systems. This is largely attributed to the high development costs related to the strict safety and reliability requirements of the regulatory systems, as already discussed [3]. Regarding the development of the systems, it is highlighted that it requires an interdisciplinary approach to deal effectively with both clinical and engineering aspects. It is important that surgeons, which are the actual end-users of the technology, are involved from the development to the marketing stage of the systems. At the same time, the technical complexity of telerobotic systems requires specialized engineering expertise.

The success of robotic surgery is based on the effective interaction between the surgeon and the robotic technology. In the case of telerobotic systems this becomes more complex given the telepresence requirements. In that respect, human factors emerge as a most critical component to ensure safer, more usable, and effective devices that will allow for their full potential to be exploited. As a result, human factors must be an integral part of the design of any telerobotic device. Of relevance is also the development of user-interfaces that provide adequate information and effective control while avoiding the display of overwhelming information to the operator. A key enhancement, expected to have a dominant impact in telepresence is the use of haptics, allowing the operating physician to sense the forces applied by the manipulation system in the remote environment [125–127]. Another issue that needs to be considered within the sphere of human factors is the fact that implementation of robotically assisted telemedicine decreases human interaction between the healthcare professionals and patients. This may increase the possibility of errors.

Software tools play an increasingly important role in telesurgery by supporting the operator actions and facilitating decision making. Preoperative planning tools often analyze imaging information and present the operator with optimal courses of action (e.g., best needle insertion path). Image-guided surgery uses images for anatomy and instrument visualization, intervention planning, as well as navigation [127–129]. Ongoing developments in the field of telesurgery also focus on vision systems, which is an essential element for guidance. Apart from camera systems, guidance has already been extended to other visualization methods, which creates new opportunities for telerobotics. Imaging information may be pre-operative but also intra-operative, and such processes range from image acquisition and image reconstruction, to image registration, and image fusion. Recent advances allow fusing intra-operative images with 3D patient-specific models constructed using pre-operative information [130]. Moreover, merging imaging intra-operative information acquired from different imaging modalities (e.g., MRI and ultrasound) for improving visualization is another possibility that can be further exploited [131–134]. In terms of medical telerobotics the transfer of imaging information through telecommunication networks is of particular importance. Toward this direction, telemedicine and tele-manipulation have become feasible capitalizing advances in telecommunications that allow reliable transmission of large amounts of data with acceptable delay, as required for the control of a master–slave system and effectiveness of the man-in-the-loop operation.

Advances in m-health medical/ultrasound video communication [35, 135] and telerobotic systems have been primarily driven by associated progresses in communication networks and video compression technologies. Increased data transfer rates facilitated by new mobile cellular networks generations over the past two decades, allowed a transition from biomedical signal to image and video communications, and then from low-bit rate video of limited clinical capacity to higher diagnostic quality medical video.

The latest 4G and beyond wireless networks deployment together with the new high-efficiency video coding (HEVC) standard [136], is expected to play a decisive role towards wider adoption in standard clinical practise. New telerobotic systems are envisioned that can compete standard in-hospital examinations. The usage of medical video communication at the clinically acquired frame rate and resolution that can be qualitatively transmitted in low delay without compromising clinical quality is the cornerstone of such advancements. Over the years, wireless networks’ security issues vulnerability has been also considerably improved [137, 138]. However, this is still an active area of research that is expected to draw significant research attention in the immediate future, as this is a matter greatly affecting adoption of telerobotics in clinical practice.

Moral as well as legal concerns need to be effectively addressed before a wider use of telerobotics is possible. Transmission of information over communication networks raises issues regarding the protection of patients’ privacy and thus needs to be regulated. Responsibility for complications during a telerobotic procedure is another delicate issue to be formally addressed. The fact that a robotically-assisted intervention can be recorded creates additional liability concerns. From a practical point of view, following unexpected technical problems or clinical complications that are likely to emerge during a robotic telesurgery (e.g., accidental injury of tissue or organs, bleeding) it may become necessary to switch to a manual method. Technological and procedural provisions should allow for a safe and timely transition, documented in relevant protocols.

In terms of the robotic manipulation system, a key issue is the safety of operation [138]. Approaches to safety of operation for medical robotics differ considerably from their industrial counterparts, which include operation in fenced workcells, and more recently other active measures (e.g., light curtains). Teleoperated medical robotics are safety-critical devices and there exist other, stricter requirements regarding their control (e.g., redundant sensing). Efficient medical telerobotic operations linked with the ever increasing application space necessitates the development of application-specific robots and instruments (end-tools). Of vital importance and a key design characteristic is the dexterity of the instruments, which is directly related to the degrees-of-freedom of the kinematic mechanisms.

Control stability/robustness present technical difficulties originating from the remote (long-distance) data transmission. Auxiliary control functions already implemented on surgical robots are expected to provide significant enhancements to the efficiency of long-distance medical telerobotics and reduce the burden on the operating physician who is confronted with the distance obstacle. Particularly important among them is the biomotion compensation which provides the system with the ability to track the motion of organs and tissue. This constitutes the operation safer and more comfortable for the physician.

Wide applicability of telesurgery will not only depend on the existence of the technology but also on the availability of trained physicians. Telesurgery requires specialized skills compared to traditional methods and it is essential that medical schools are equipped with such technologies to appropriately train physicians. Here, the cost of the equipment again emerges as a major obstacle but to some extent simulation tools can remedy the situation [6]. Another available option—inherent to the nature of the system—is telementoring [139, 140]. An experienced physician can play the preceptor’s role to other physician without having to relocate or travel. At the same time telementoring improves the confidence levels of novice physicians and their willingness to pursue a non-traditional method such as telesurgery. Existing possibilities include the use of systems with a dual control console configuration (e.g., da Vinci) to enable training (teaching/mentoring) or collaborative surgery. In fact, the two control consoles may be installed at separate geographic locations. Educational and other prerequisites before a surgeon can utilize the telerobotic technology need to be formally established. Legal regulation is particularly important in order to prevent unauthorized service providers in this sector. Beyond education, academic institutions may also play an important role in relevant research. Medical telerobotics is an emerging field and collaborative efforts with universities are essential so as to develop new, commercializable technologies.

## Conclusions

Medical telerobotic is an emerging field expected to have a significant impact on healthcare. Indicative of its potential is the fact that telerobotics has already been considered for a wide range of applications and medical disciplines, which is apparent from the present review. It is also noticeable the fact that the large majority of existing systems have been short-distance ones and the potential of operating them remotely remains largely unexploited. This fact signifies that the ultimate goal of employing robotic manipulation in telemedicine, in order to provide specialized medical services remotely, has not been accomplished yet nor the full potential of telerobotic has been unleashed.

Future developments in the field of telerobotics will require addressing specific clinical as well as technological challenges following an interdisciplinary approach. The involvement of physicians in the development stage of telerobotic systems and emphasis on clinical studies are keys to producing clinically-oriented solutions. Technological challenges are related to three basic enabling technologies: robotic manipulation, vision systems and telecommunications. For the transition from short-distance to long-distance telerobotic systems a major role depends on the telecommunications links. Therefore, latest telecommunication technologies should be embraced to ensure efficient, reliable and safe transmission of data.

Prior to commercialization, prototype experimental systems have to be refined to become more usable, safe, reliable, elegant and appealing to users but also meet the regulatory requirements. Human factors play a significant role towards that direction. To effectively practice telerobotics, physicians will be required to obtain new skills and relevant training should also be considered in medical schools. Telementoring is an option to be considered towards this direction which is compatible and can be built in the telerobotic technology.

Ultimately, clinical acceptance will depend on the ability of the telerobotic technology to demonstrate measurable benefits to the healthcare system based on improved clinical results, efficiency of operations, and cost effectiveness. Quality of care in surgery is often evaluated on the basis of success rates, complications that may occur, and length of hospitalization. However, the most significant benefit expected from the use of long-distance telerobotic systems will be the ability to provide specialized medical services (diagnostic or therapeutic) to remote or isolated areas while avoiding physician/patient travel costs and inconveniences. Important steps towards this direction have already been achieved but the true potential of medical telerobotic remains largely unexploited.

## Abbreviations

4G: 4th Generation
CCD: charge coupled device
CT: computed tomography
DOF: degrees-of-freedom
ENT: ear nose throat
FDA: Food and Drug Administration
HEVC: high efficiency video coding standard
LAN: local area network
MIS: minimally invasive surgery
MRI: magnetic resonance imaging
NOTES: natural orifice transluminal endoscopic surgery
QSR: Quality System Regulation
SPA: single port access
TATRC: Telemedicine and Advanced Technology Research Center
TCP: transmission control protocol
UDP: user datagram protocol
US: ultrasound
